# Functional adaptations in the cecal and colonic metagenomes associated with the consumption of transglycosylated starch in a pig model

**DOI:** 10.1186/s12866-019-1462-2

**Published:** 2019-05-02

**Authors:** Barbara U. Metzler-Zebeli, Monica A. Newman, Dietmar Grüll, Qendrim Zebeli

**Affiliations:** 1 Department for Farm Animals and Veterinary Public Health , Institute of Animal Nutrition and Functional Plant Compounds, University of Veterinary Medicine Vienna, 1210 Vienna, Austria; 2 Agrana Research & Innovation Center GmbH, 3430 Tulln, Austria

**Keywords:** Transglycosylated starch, Pig, Large intestine, Metagenome, Starch catabolism, Microbe-host-interaction

## Abstract

**Background:**

Both phylogeny and functional capabilities within the gut microbiota populations are of great importance for influencing host health. As a novel type of resistant starch, transglycosylated starch (TGS) modifies the microbial community and metabolite profiles along the porcine gut, but little is known about the related functional adaptations in key metabolic pathways and their taxonomic identity.

**Results:**

Metagenomic sequencing was used to characterize the functional alterations in the cecal and colonic microbiomes of growing pigs fed TGS or control starch (CON) diets for 10 days (*n* = 8/diet). Bacterial communities were clearly distinguishable at taxonomic and functional level based on the dietary starch, with effects being similar at both gut sites. Cecal and colonic samples from TGS-fed pigs were enriched in *Prevotella*, *Bacteroides*, *Acidaminoccus* and *Veillonella*, whereas *Treponema*, *Ruminococcus,* and *Aeromonas* declined at both gut sites compared to CON-fed pigs (log_2_ fold change > ±1; *p* < 0.001 (*q* < 0.05)). This was associated with increased enzymatic capacities for amino acid metabolism, galactose, fructose and mannose metabolism, pentose and glucuronate interconversions, citrate cycle and vitamin metabolism for samples from TGS-fed pigs. However, TGS-fed pigs comprised fewer reads for starch and sucrose metabolism and genetic information processing. Changes in key catabolic steps were found to be the result of changes in taxa associated with each type of starch. Functional analysis indicated steps in the breakdown of TGS by the action of α- and β-galactosidases, which mainly belonged to *Bacteroides* and *Prevotella*. Reads mapped to alpha-amylase were less frequent in TGS- compared to CON-fed pigs, with the major source of this gene pool being *Bacillus*, *Aeromonas* and *Streptococcus*. Due to the taxonomic shifts, gene abundances of potent stimulants of the mucosal innate immune response were altered by the starches. The cecal and colonic metagenomes of TGS-fed pigs comprised more reads annotated in lipopolysaccharides biosynthesis, whereas they became depleted of genes for flagellar assembly compared to CON-fed pigs.

**Conclusions:**

Metagenomic sequencing revealed distinct cecal and colonic bacterial communities in CON- and TGS-fed pigs, with strong discrimination among samples by functional capacities related to the respective starch in each pig’s diet.

**Electronic supplementary material:**

The online version of this article (10.1186/s12866-019-1462-2) contains supplementary material, which is available to authorized users.

## Background

The gut microbiota has a fundamental impact on host metabolism, immune functions and physiology with implications for gut and systemic health [1], whereby the diet of the host is their most important source of nutrients [2]. Poor nutrition will therefore not only impair the metabolic health of the host but lead to significant changes in the gut microbiota normobiosis [1]. Despite this awareness, our knowledge about the concurrent changes in the functional attributes in the bacterial microbiome is still limited.

Due to their high fermentability, there is growing interest to increase the proportion of resistant starch (RS) in human and animal diets for their potential prebiotic abilities. Resistant starches are long known for their blood glucose lowering effect because they resist digestion by mammalian enzymes in the small intestine [3]. Consequently, they become available to a large extent to the microbiota in the hindgut, altering the microbial composition and their metabolic activities including increased short-chain fatty acid (SCFA) production [4–6]. Currently, there are five types of RS of which type 2 and 3 are the best researched RS. In recent years, the development of low digestible chemically modified starches (RS type 4) has been promoted. These starches may fulfil a dual functionality by enhancing texture and rheological properties of processed foods [7] and, if low digestible, by increasing the daily dietary fiber intake. Especially, the modification of the chemical structure in chemically modified starches, such as cross-linking, esterification or tranglycosylation, may alter the pathways used by the microbes for the hydrolysis of the starch probably due to the promotion of other bacteria as with RS types 2 and 3 [4, 6, 8, 9].

Both phylogeny and functional capabilities within the gut microbiota populations are of great importance as they both impact host physiology and health [10]. Whereas progress has been made in regard to the knowledge of RS-related phylogenetic shifts, our current understanding of the functional adaptations in the gut microbiome due to RS consumption is still in its infancy. In-depth sequencing approaches using 16S rRNA gene sequencing were extremely valuable to characterize the effect of the various RS types on the gastrointestinal bacterial microbiome [6, 8, 9]. Based on 16S rRNA gene analysis, we could recently show for a transglycosylated starch (TGS) to largely alter the gastric, ileal, cecal and colonic bacterial microbiomes of growing pigs, with the qualitative changes being almost identical at the different gut sites [9]. Moreover, by identifying the most influential bacterial genera and medium-chain fatty acids and SCFA, the TGS induced a different bacterial signature on mucosal signaling compared to the waxy cornstarch fed as control [11]. The present objective was therefore to elucidate the changes in the predicted functional composition of the bacterial microbiome in the cecum and colon of growing pigs after TGS consumption using shotgun metagenome sequencing. We hypothesized that the alterations in the functional abilities that accompany the taxonomic changes in the cecum and colon of pigs will reveal the functional pathways required to utilize TGS and to interact with the host animal as part of the complex relationship between diet, microbial function and host physiology and metabolism.

## Results

All pigs were clinically healthy throughout the experiment.

### General characteristics of the metagenomes

To investigate alterations in the predicted functional composition of the bacterial microbiome in the cecum and colon of growing pigs after TGS consumption, DNA was extracted from intestinal samples of pigs fed either the TGS (*n* = 8) or control (CON) diet (*n* = 8). Illumina NextSeq sequencing of multiplexed 150-bp libraries using a high-output, single-end protocol was employed to describe the taxonomic and functional microbial composition.

Shotgun metagenomic sequencing resulted in on average 13,206,140 and 14,094,761 reads in cecal and colonic samples, with a total of 1,973,401,005 and 2,105,585,724 bp, respectively, and an average read length of 149 bp at both gut sites. After the quality control, 9,691,736 and 9,697,270 proteins were predicted for cecal and colonic samples and 72.5 and 69.4% of the total reads in cecal and colonic samples were annotated as proteins functionally assigned, respectively.

### Comparison of taxonomic profiles reshaped by diet

For the domain level, the microbiota profiles in the cecal and colonic samples generated by MG-RAST were predominated by *Eubacteria,* whereas archaeal counts only amounted to approximately 1% at both gut sites (Table 1). At phylum level, the cecal and colonic samples were dominated by *Bacteroidetes*, *Firmicutes* and *Proteobacteria* (Additional file 1: Figure S1). Within the archaeal community, *Euryarchaeota* (~ 95%) clearly predominated, followed by *Crenaerchaeota* (~ 4.0%) and *Thaumaarchaeota* (~ 0.5%) with similar abundances at both gut sites.

**Table 1:** Taxonomic profile of cecal and colonic samples of CON- and TGS-fed pigs at domain level

Domain	Mean^a^	log_2_ fold change	SE^b^	*p* value	*q* value^‡^
Cecum					
*Archaea*	24,714	−0.018	0.067	0.789	0.930
*Bacteria*	2,897,040	0.729	0.206	< 0.001	0.003
Colon					
*Archaea*	34,107	−0.128	0.076	0.091	0.135
*Bacteria*	2,917,299	0.533	0.125	< 0.001	< 0.001

^‡^False discovery rate (Benjamini-Hochberg) corrected *p* value

^a^Normalized reads (hit counts). Only the most abundant genera (> 0.01% of all reads) that were altered by the dietary starch source are presented (*n* = 8 per diet and gut site)

^b^Standard error of the log_2_fold change

The TGS diet mainly shaped the cecal and colonic communities of the domain *Bacteria* (Table 1; Additional file 1: Figure S1 and S2), which increased by 0.73 and 0.53 log_2_ folds in cecal and colonic digesta, respectively, of TGS-fed pigs compared to pigs receiving the control (CON) diet (*p* < 0.05 (*q* < 0.01)). In contrast, *Archaea* counts were similar between the feeding groups at both gut sites. In both gut segments (Additional file 1: Figure S1a and S1b), TGS-fed pigs comprised more *Bacteroidetes* and *Actinobacteria*, but less *Spirochaetes* counts compared to CON-fed pigs (*p* < 0.05 (*q* < 0.10)). With respect to archaeal phyla, only the *Crenaerchaeota* decreased in cecal samples of TGS- compared to CON-fed pigs by − 0.47 log_2_ folds (*p* = 0.007 (*q* = 0.030)). Dietary starch-related bacterial community profiles were shown in the distance matrices at phylum level (Additional file 1: Figure S1c and S1d). Of the predominant bacterial genera (Additional file 1: Figure S2a and S2b), the TGS diet enriched the cecal and colonic communities with *Prevotella*, *Bacteroides*, *Acidaminococcus*, *Veillonella* (*p* < 0.001 (*q* < 0.05)) and *Lactobacillus* (*p* < 0.05 (*q* < 0.1)) compared to the CON diet. By contrast, pigs fed the TGS diet comprised less reads of the predominant genera *Eubacterium*, *Treponema, Ruminococcus* and *Butyrivibrio* in cecal samples as well as of *Clostridium* and *Ruminococcus* in colonic samples compared to CON-fed pigs (*p* < 0.05 (*q* < 0.1)).

### Functional composition of the metagenome reshaped by diet

**Fig. 1:**
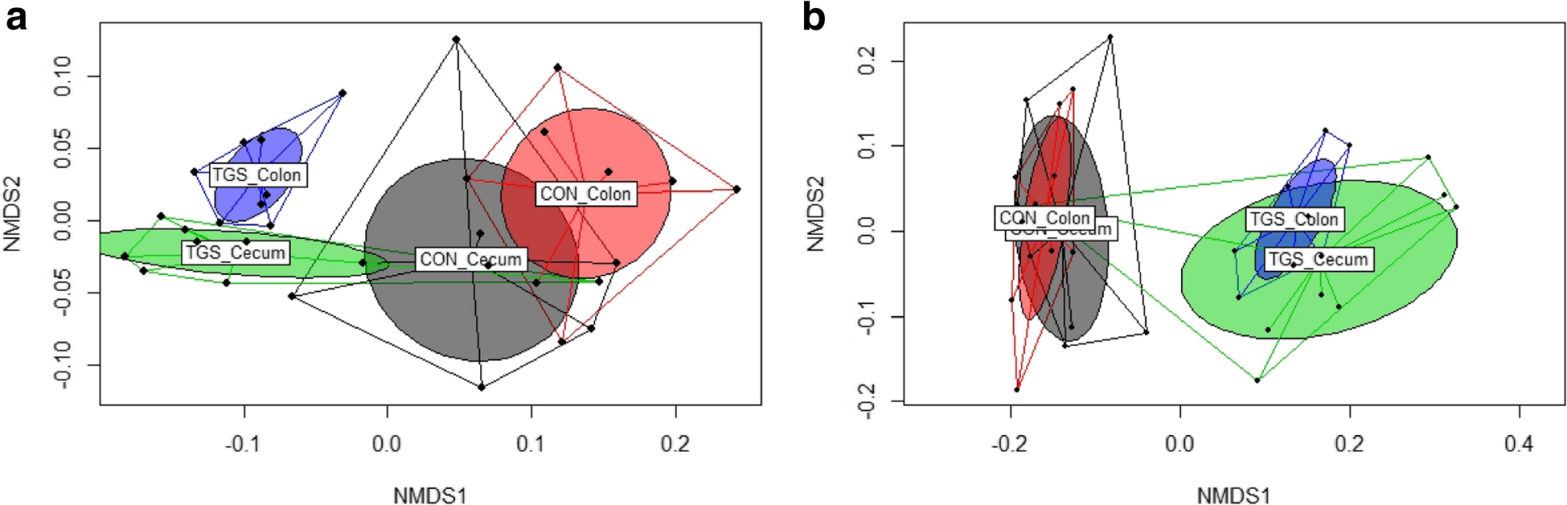
Two-dimensional non-parametric multidimensional scaling (NMDS) ordination plots of predicted bacterial KEGG pathways (KEGG orthology (KO) level 3) and functions within the KEGG pathway ‘carbohydrate metabolism’ in cecal and colonic samples of control (CON) and transglycosylated starch (TGS)-fed pigs (*n* = 8 per diet and gut site). The NMDS plots were generated using Bray-Curtis distance metric between dietary starches. Each dot represents an individual samples; the circles indicate the SD. **a** KO level 3 pathways in cecal and colonic samples (stress = 0.1103); and **b** ‘carbohydrate metabolism’-related functions in cecal and colonic samples (stress = 0.0998)

To identify the major changes in the functional composition, we linked the genes in the present metagenomes to Kyoto Encyclopedia of Genes and Genomes (KEGG) orthology (KO) classes and obtained the functional profiles of the metagenomes [12]. The functional profiles were first compared by non-parametric multidimensional scaling (NMDS), using reads that could be mapped to functional annotations (Fig. 1). The NMDS ordinal plots for KO level 3 and KO functions within ‘carbohydrate metabolism’ showed distinct clustering of the samples according to the two diets, whereas overlaps existed between gut sites (Fig. 1a and b). Nevertheless, when analyzing the single functions, the functional changes caused by the TGS product were qualitatively similar in cecal and colonic samples. Of the 62 KO level 3 pathways that were differently abundant in the cecal samples, 31 pathways were enriched and 31 pathways were less frequent with the TGS compared to the CON diet (Table 2). In colonic digesta, 94 KO level 3 pathways were differently abundant in TGS- and CON-fed pigs, 42 were more and 52 were less abundant with the TGS compared to the CON diet (Table 3). Differences between samples were detected for reads that were functionally annotated as being involved in ‘amino acid metabolism’, ‘carbohydrate metabolism’, ‘biosynthesis of other secondary metabolites’, ‘glycan biosynthesis and metabolism’, ‘membrane transport’, ‘metabolism of cofactors and vitamins’, and ‘transport and catabolism’ as well as related to the ‘genetic information processing’, irrespective of the gut site. Of interest, reads related to the citrate cycle and vitamin synthesis were more frequent, whereas genes related to transcription and translation declined in both cecal and colonic samples of TGS- compared to CON-fed pigs. Also of note, reads annotated as methane metabolism and thus related to archaeal metabolism declined in cecal and colonic samples of TGS- compared to CON-fed pigs.

**Table 2:** Selected KEGG pathways (KEGG orthology level 3) in cecal samples being differently enriched in CON- and TGS-fed pigs

KEGG pathway	Mean^a^	log_2_ fold change	SE^b^	*p* value	*q* value^‡^
00633 Nitrotoluene degradation [PATH:ko00633]	389	1.982	0.375	< 0.001	< 0.001
00983 Drug metabolism - other enzymes [PATH:ko00983]	1046	1.772	0.353	< 0.001	< 0.001
00940 Phenylpropanoid biosynthesis [PATH:ko00940]	9129	1.613	0.213	< 0.001	< 0.001
02060 Phosphotransferase system (PTS) [PATH:ko02060]	6289	1.040	0.217	< 0.001	< 0.001
00130 Ubiquinone and other terpenoid-quinone biosynthesis [PATH:ko00130]	3982	0.969	0.224	< 0.001	< 0.001
00040 Pentose and glucuronate interconversions [PATH:ko00040]	14,629	0.803	0.191	< 0.001	< 0.001
04141 Protein processing in endoplasmic reticulum [PATH:ko04141]	803	0.789	0.193	< 0.001	< 0.001
00052 Galactose metabolism [PATH:ko00052]	20,395	0.739	0.125	< 0.001	< 0.001
00051 Fructose and mannose metabolism [PATH:ko00051]	17,994	0.673	0.126	< 0.001	< 0.001
00730 Thiamine metabolism [PATH:ko00730]	9877	0.607	0.125	< 0.001	< 0.001
02010 ABC transporters [PATH:ko02010]	49,093	−0.413	0.075	< 0.001	< 0.001
03013 RNA transport [PATH:ko03013]	1493	−0.453	0.112	< 0.001	< 0.001
04122 Sulfur relay system [PATH:ko04122]	5609	−0.976	0.197	< 0.001	< 0.001
00360 Phenylalanine metabolism [PATH:ko00360]	2011	−1.286	0.311	< 0.001	< 0.001
02040 Flagellar assembly [PATH:ko02040]	7812	−1.370	0.269	< 0.001	< 0.001
00380 Tryptophan metabolism [PATH:ko00380]	255	−1.651	0.294	< 0.001	< 0.001
00531 Glycosaminoglycan degradation [PATH:ko00531]	391	1.077	0.275	< 0.001	0.001
00860 Porphyrin and chlorophyll metabolism [PATH:ko00860]	7948	0.594	0.152	< 0.001	0.001
00680 Methane metabolism [PATH:ko00680]	4678	−0.375	0.096	< 0.001	0.001
00630 Glyoxylate and dicarboxylate metabolism [PATH:ko00630]	3354	−0.418	0.108	< 0.001	0.001
03070 Bacterial secretion system [PATH:ko03070]	24,985	−0.367	0.104	< 0.001	0.002
00500 Starch and sucrose metabolism [PATH:ko00500]	18,633	−0.245	0.075	0.001	0.006
04142 Lysosome [PATH:ko04142]	3227	0.593	0.187	0.002	0.007
00350 Tyrosine metabolism [PATH:ko00350]	2333	−0.932	0.294	0.002	0.007
00511 Other glycan degradation [PATH:ko00511]	2316	0.665	0.214	0.002	0.008
04151 PI3K-Akt signaling pathway [PATH:ko04151]	5164	−0.822	0.267	0.002	0.009
00590 Arachidonic acid metabolism [PATH:ko00590]	829	1.026	0.351	0.003	0.014
00300 Lysine biosynthesis [PATH:ko00300]	23,314	0.136	0.047	0.004	0.014
00510 N-Glycan biosynthesis [PATH:ko00510]	918	0.545	0.202	0.007	0.024
00564 Glycerophospholipid metabolism [PATH:ko00564]	5568	0.154	0.057	0.007	0.024
00010 Glycolysis / Gluconeogenesis [PATH:ko00010]	23,481	−0.160	0.059	0.007	0.024
00061 Fatty acid biosynthesis [PATH:ko00061]	13,010	−0.165	0.061	0.007	0.024
00330 Arginine and proline metabolism [PATH:ko00330]	26,554	0.284	0.106	0.007	0.025
00071 Fatty acid metabolism [PATH:ko00071]	133	−1.077	0.423	0.011	0.036
00900 Terpenoid backbone biosynthesis [PATH:ko00900]	12,834	0.216	0.086	0.012	0.037
00020 Citrate cycle (TCA cycle) [PATH:ko00020]	15,173	0.415	0.171	0.015	0.045
03020 RNA polymerase [PATH:ko03020]	29,030	−0.385	0.159	0.015	0.045
02030 Bacterial chemotaxis [PATH:ko02030]	8461	−0.680	0.278	0.015	0.045
03060 Protein export [PATH:ko03060]	1992	0.193	0.083	0.020	0.056
00520 Amino sugar and nucleotide sugar metabolism [PATH:ko00520]	18,729	0.147	0.064	0.022	0.062
00280 Valine leucine and isoleucine degradation [PATH:ko00280]	5486	−0.240	0.108	0.026	0.067
00785 Lipoic acid metabolism [PATH:ko00785]	167	−0.642	0.287	0.025	0.067
00030 Pentose phosphate pathway [PATH:ko00030]	12,994	−0.128	0.058	0.027	0.068
00750 Vitamin B6 metabolism [PATH:ko00750]	4284	0.280	0.131	0.033	0.077
00540 Lipopolysaccharide biosynthesis [PATH:ko00540]	10,816	0.615	0.292	0.035	0.081
00908 Zeatin biosynthesis [PATH:ko00908]	2398	0.174	0.085	0.040	0.091
04146 Peroxisome [PATH:ko04146]	6168	−0.179	0.088	0.041	0.091
00053 Ascorbate and aldarate metabolism [PATH:ko00053]	334	0.498	0.250	0.046	0.099
03018 RNA degradation [PATH:ko03018]	30,822	−0.183	0.092	0.046	0.099

^‡^False discovery rate (Benjamini-Hochberg) corrected *p* value

^a^Normalized reads (hit counts). Only the most abundant KEGG pathways (> 0.01% of all reads) that were altered by the dietary starch source are presented (*n* = 8 per diet)

^b^Standard error of the log_2_fold change

**Table 3:** Selected KEGG pathways (KEGG orthology level 3) in colonic samples being differently enriched in CON- and TGS-fed pigs

KEGG pathway	Mean^a^	log_2_ fold change	SE^b^	*p* value	*q* value^‡^
00633 Nitrotoluene degradation [PATH:ko00633]	661	2.575	0.351	< 0.001	< 0.001
00983 Drug metabolism - other enzymes [PATH:ko00983]	818	1.910	0.160	< 0.001	< 0.001
00531 Glycosaminoglycan degradation [PATH:ko00531]	397	1.868	0.276	< 0.001	< 0.001
02060 Phosphotransferase system (PTS) [PATH:ko02060]	9299	1.739	0.228	< 0.001	< 0.001
00940 Phenylpropanoid biosynthesis [PATH:ko00940]	8963	1.542	0.148	< 0.001	< 0.001
00130 Ubiquinone and other terpenoid-quinone biosynthesis [PATH:ko00130]	3636	1.510	0.145	< 0.001	< 0.001
00710 Carbon fixation in photosynthetic organisms [PATH:ko00710]	202	1.422	0.373	< 0.001	< 0.001
00590 Arachidonic acid metabolism [PATH:ko00590]	922	1.336	0.321	< 0.001	< 0.001
00910 Nitrogen metabolism [PATH:ko00910]	1675	1.152	0.262	< 0.001	< 0.001
00740 Riboflavin metabolism [PATH:ko00740]	4762	1.040	0.199	< 0.001	< 0.001
00511 Other glycan degradation [PATH:ko00511]	2787	1.016	0.174	< 0.001	< 0.001
00051 Fructose and mannose metabolism [PATH:ko00051]	16,391	0.746	0.089	< 0.001	< 0.001
04141 Protein processing in endoplasmic reticulum [PATH:ko04141]	784	0.728	0.143	< 0.001	< 0.001
00052 Galactose metabolism [PATH:ko00052]	21,809	0.713	0.091	< 0.001	< 0.001
04142 Lysosome [PATH:ko04142]	2980	0.683	0.153	< 0.001	< 0.001
00860 Porphyrin and chlorophyll metabolism [PATH:ko00860]	10,480	0.647	0.102	< 0.001	< 0.001
00040 Pentose and glucuronate interconversions [PATH:ko00040]	13,379	0.593	0.101	< 0.001	< 0.001
00750 Vitamin B6 metabolism [PATH:ko00750]	3823	0.559	0.127	< 0.001	< 0.001
00281 Geraniol degradation [PATH:ko00281]	614	0.549	0.144	< 0.001	< 0.001
00020 Citrate cycle (TCA cycle) [PATH:ko00020]	16,331	0.533	0.089	< 0.001	< 0.001
00730 Thiamine metabolism [PATH:ko00730]	8664	0.522	0.097	< 0.001	< 0.001
00790 Folate biosynthesis [PATH:ko00790]	3122	0.507	0.124	< 0.001	< 0.001
00400 Phenylalanine tyrosine and tryptophan biosynthesis [PATH:ko00400]	17,524	0.386	0.094	< 0.001	< 0.001
00330 Arginine and proline metabolism [PATH:ko00330]	27,975	0.296	0.058	< 0.001	< 0.001
00340 Histidine metabolism [PATH:ko00340]	15,847	0.252	0.048	< 0.001	< 0.001
03018 RNA degradation [PATH:ko03018]	31,058	−0.206	0.052	< 0.001	< 0.001
03010 Ribosome [PATH:ko03010]	59,767	−0.279	0.066	< 0.001	< 0.001
00010 Glycolysis / Gluconeogenesis [PATH:ko00010]	25,589	−0.307	0.043	< 0.001	< 0.001
00970 Aminoacyl-tRNA biosynthesis [PATH:ko00970]	96,501	−0.377	0.066	< 0.001	< 0.001
03070 Bacterial secretion system [PATH:ko03070]	26,474	−0.429	0.079	< 0.001	< 0.001
03020 RNA polymerase [PATH:ko03020]	30,568	−0.656	0.072	< 0.001	< 0.001
00310 Lysine degradation [PATH:ko00310]	788	−0.991	0.220	< 0.001	< 0.001
04151 PI3K-Akt signaling pathway [PATH:ko04151]	4509	−1.058	0.083	< 0.001	< 0.001
00360 Phenylalanine metabolism [PATH:ko00360]	2719	−1.103	0.149	< 0.001	< 0.001
04122 Sulfur relay system [PATH:ko04122]	7389	−1.137	0.086	< 0.001	< 0.001
00350 Tyrosine metabolism [PATH:ko00350]	3212	− 1.404	0.164	< 0.001	< 0.001
00785 Lipoic acid metabolism [PATH:ko00785]	187	−1.562	0.244	< 0.001	< 0.001
00380 Tryptophan metabolism [PATH:ko00380]	304	−1.595	0.181	< 0.001	< 0.001
00791 Atrazine degradation [PATH:ko00791]	196	−2.087	0.374	< 0.001	< 0.001
03040 Spliceosome [PATH:ko03040]	203	−2.108	0.292	< 0.001	< 0.001
00660 C5-Branched dibasic acid metabolism [PATH:ko00660]	142	−4.390	0.563	< 0.001	< 0.001
00540 Lipopolysaccharide biosynthesis [PATH:ko00540]	9910	0.965	0.261	0.000	0.001
02010 ABC transporters [PATH:ko02010]	65,587	−0.243	0.070	0.001	0.001
00500 Starch and sucrose metabolism [PATH:ko00500]	22,311	−0.256	0.073	< 0.001	0.001
03420 Nucleotide excision repair [PATH:ko03420]	23,037	−0.270	0.078	0.001	0.001
00680 Methane metabolism [PATH:ko00680]	5869	−0.359	0.099	< 0.001	0.001
00071 Fatty acid metabolism [PATH:ko00071]	136	−1.551	0.426	< 0.001	0.001
00230 Purine metabolism [PATH:ko00230]	49,811	−0.134	0.040	0.001	0.002
00640 Propanoate metabolism [PATH:ko00640]	530	−0.546	0.165	0.001	0.002
00510 N-Glycan biosynthesis [PATH:ko00510]	785	0.460	0.148	0.002	0.004
00900 Terpenoid backbone biosynthesis [PATH:ko00900]	13,580	0.143	0.046	0.002	0.004
00620 Pyruvate metabolism [PATH:ko00620]	22,435	−0.192	0.063	0.002	0.005
03060 Protein export [PATH:ko03060]	1837	0.318	0.107	0.003	0.006
00250 Alanine aspartate and glutamate metabolism [PATH:ko00250]	69,446	0.133	0.045	0.003	0.007
00260 Glycine serine and threonine metabolism [PATH:ko00260]	42,580	−0.110	0.039	0.005	0.009
03013 RNA transport [PATH:ko03013]	1374	−0.285	0.111	0.010	0.020
00550 Peptidoglycan biosynthesis [PATH:ko00550]	17,416	−0.145	0.059	0.014	0.027
01040 Biosynthesis of unsaturated fatty acids [PATH:ko01040]	108	−1.247	0.512	0.015	0.028
00053 Ascorbate and aldarate metabolism [PATH:ko00053]	466	0.595	0.250	0.017	0.031
03030 DNA replication [PATH:ko03030]	31,056	−0.233	0.098	0.018	0.031
00300 Lysine biosynthesis [PATH:ko00300]	23,272	0.118	0.050	0.019	0.033
00520 Amino sugar and nucleotide sugar metabolism [PATH:ko00520]	20,592	0.107	0.046	0.020	0.035
00362 Benzoate degradation [PATH:ko00362]	479	0.797	0.368	0.030	0.051
03440 Homologous recombination [PATH:ko03440]	20,390	−0.127	0.060	0.035	0.058
00440 Phosphonate and phosphinate metabolism [PATH:ko00440]	788	0.628	0.307	0.041	0.065
00561 Glycerolipid metabolism [PATH:ko00561]	5347	0.269	0.135	0.046	0.071
00290 Valine leucine and isoleucine biosynthesis [PATH:ko00290]	23,041	0.159	0.080	0.046	0.071
00270 Cysteine and methionine metabolism [PATH:ko00270]	33,967	0.118	0.061	0.055	0.083
02030 Bacterial chemotaxis [PATH:ko02030]	8408	0.622	0.327	0.057	0.085
00030 Pentose phosphate pathway [PATH:ko00030]	14,947	−0.111	0.059	0.062	0.090

^‡^ False discovery rate (Benjamini-Hochberg) corrected *p* value

^a^Normalized reads (hit counts). Only the most abundant KEGG pathways (> 0.01% of all reads) that were altered by the dietary starch source are presented (*n* = 8 per diet)

^b^Standard error of the log_2_fold change

**Fig. 2:**
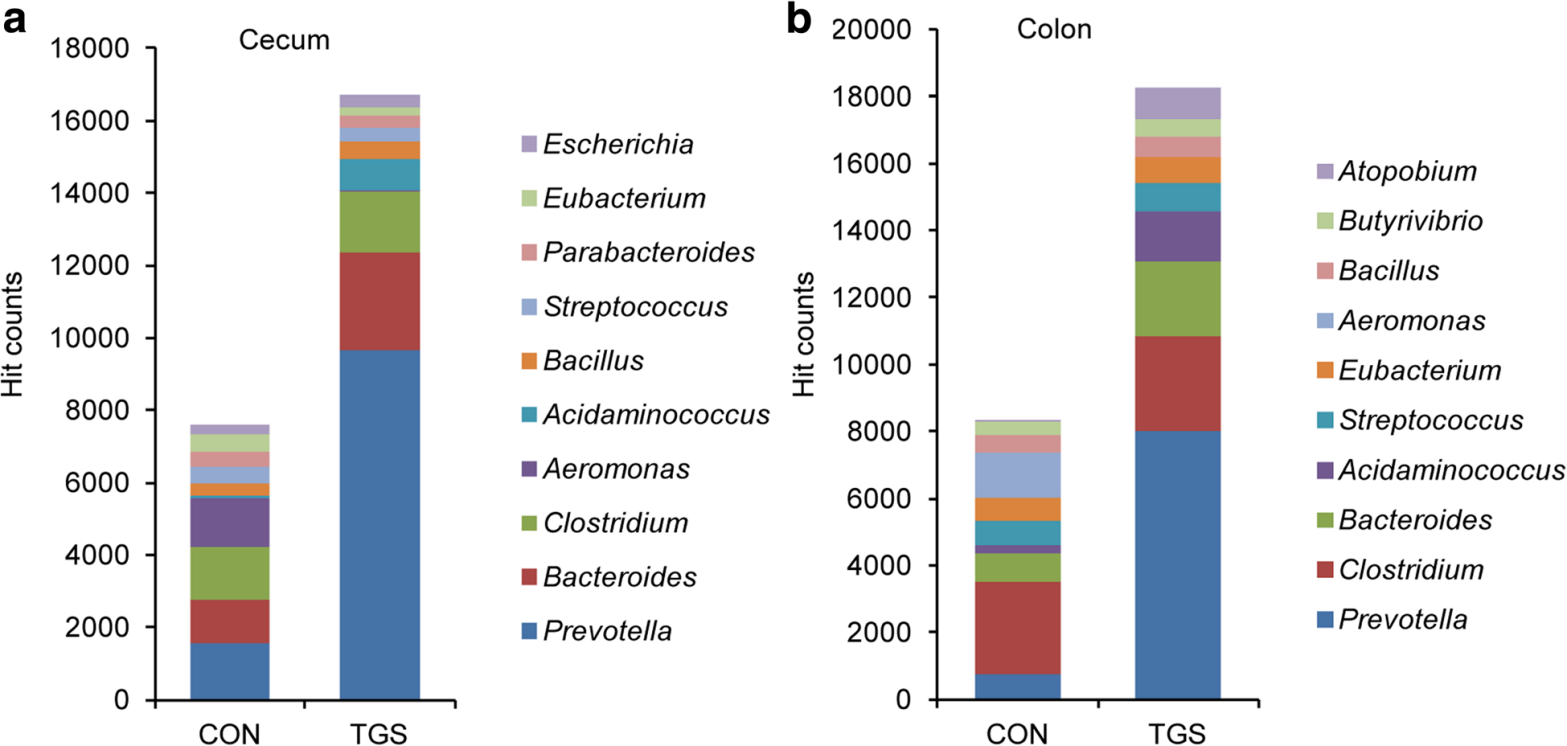
Taxonomic identity of predicted functions within the KEGG pathway ‘starch and sucrose metabolism’ in cecal (**a**) and colonic samples (**b**) of control starch (CON)- and transglycosylated starch (TGS)-fed pigs (*n* = 8 per diet and gut site). Values are presented as the mean of hit counts per dietary starch. Hit counts were normalized with DESeq2-size factors. Only the taxonomic identity of the 10 most abundant genera is presented

**Fig. 3:**
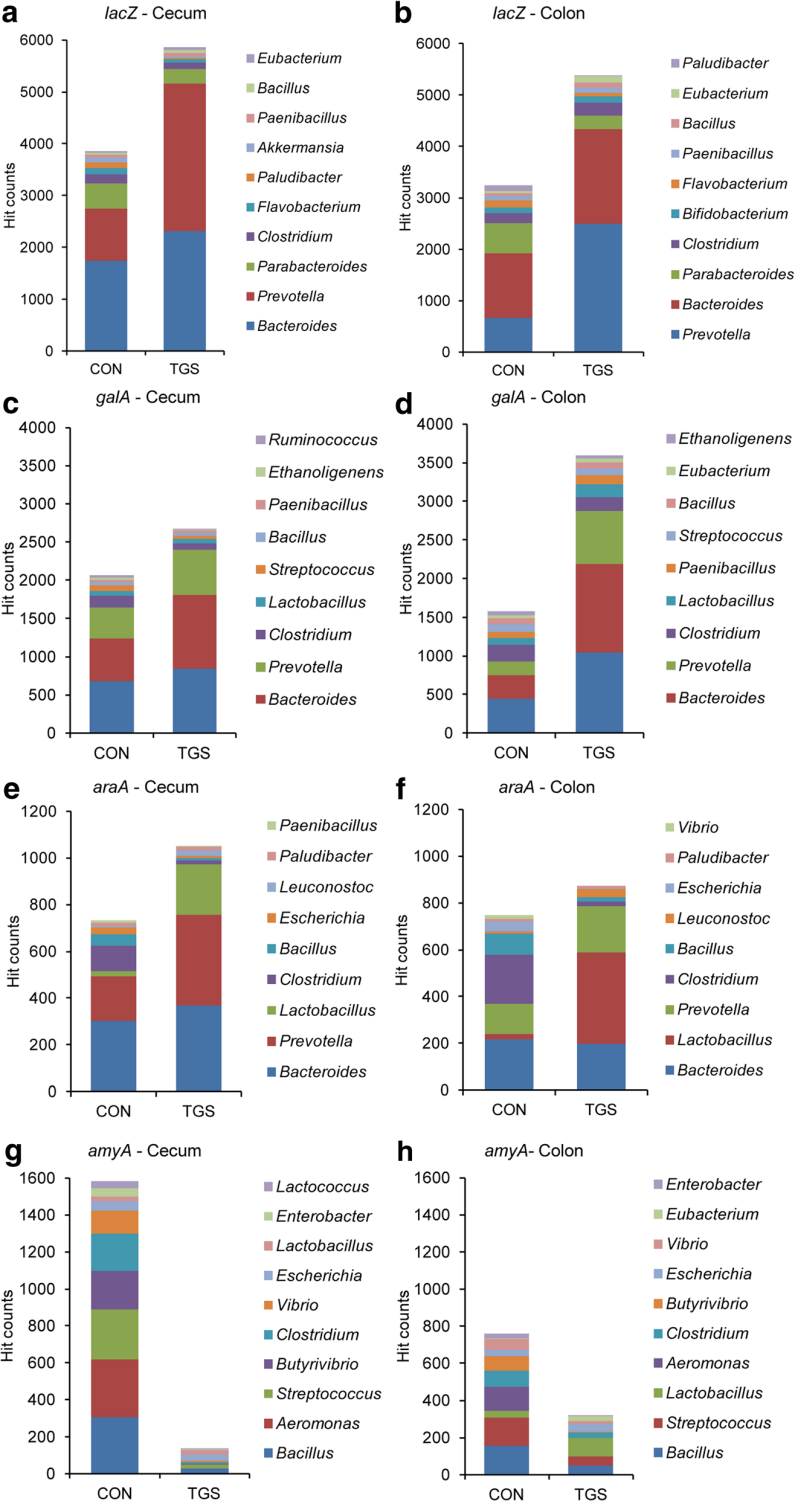
Taxonomic identity of selected predicted functions related to carbohydrate metabolism in cecal and colonic samples of control starch (CON)- and transglycosylated starch (TGS)-fed pigs (*n* = 8 per diet and gut site). **a** beta-galactosidase (*lacZ*) in cecal samples; **b** beta-galactosidase (*lacZ*) in colonic samples; **c** alpha-galactosidase (*galA*) in cecal samples; **d** alpha-galactosidase (*galA*) in colonic samples; **e** alpha-amylase (*amyA*) in cecal samples; **f** alpha-amylase (*amyA*) in colonic samples; **g** L-arabinose isomerase (*araA*) in cecal samples; and **h** L-arabinose isomerase (*araA*) in colonic samples. Values are presented as the mean of hit counts per dietary starch. Hit counts were normalized with DESeq2-size factors. Only the taxonomic identity of the most abundant genera is presented. Abundance change significance (*p* < 0.05 (false discovery rate, *q* < 0.10)) between dietary starches is presented in Additional file 1: Table S3-S6

With respect to genes mapping for ‘carbohydrate metabolism’, read abundances for key catabolic steps in the degradation of glycans and energy metabolism were altered by the TGS compared to the CON diet (Additional file 1: Tables S1 and S2). Accordingly, cecal and colonic samples of TGS-fed pigs were enriched for genes mapping for ‘galactose metabolism’, ‘fructose and mannose metabolism’, ‘pentose and glucuronate interconversions’ and ‘citrate cycle’, whereas the TGS decreased the enzymatic capacities related to ‘starch and sucrose metabolism’ compared to the CON diet at both gut sites. The bacterial contribution to the KO function ‘starch and sucrose metabolism’ largely changed with more hits being contributed by *Prevotella*, *Bacteroides* and *Acidaminococcus* in cecal and colonic samples of TGS-fed pigs (Fig. 2a and b). The screening of the functional enzymes indicated steps in the breakdown of TGS by the action of α- and β-galactosidases, fructokinase, galactokinase, arabinose isomerase and xylulokinase at both gut sites (Fig. 3a-f; Additional file 1: Tables S1-S6). Key enzymes that were mainly affected by the TGS diet within the ‘starch and sucrose metabolism’ included alpha-glucosidases and alpha-amylases. For instance, the reduced abundance of *Bacillus*, *Aeromonas*, *Streptococcus*, *Butyrivibrio*, *Clostridium* and *Vibrio* in TGS- compared to CON-fed pigs reduced the reads related to the alpha-amylase gene *amyA* (Fig. 3g and h; Additional file 1: Table S5).

**Fig. 4:**
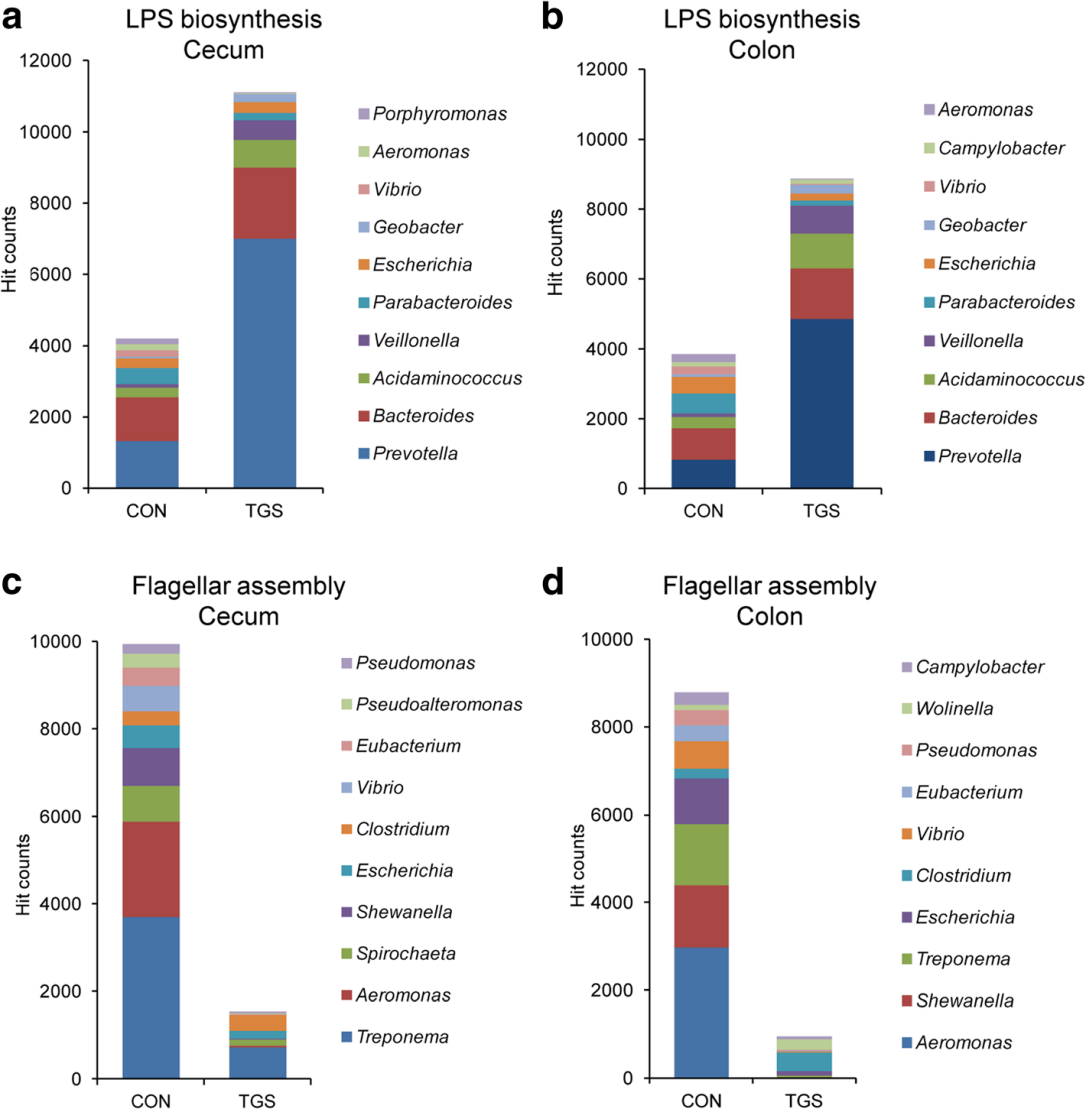
Taxonomic identity of predicted functions for lipopolysaccharide biosynthesis and flagellar assembly in cecal and colonic samples of control starch (CON) and transglycosylated starch (TGS)-fed pigs (*n* = 8 per diet and gut site). **a** gene abundance related to lipopolysaccharide biosynthesis in cecal samples; **b** gene abundance related to lipopolysaccharide biosynthesis in colonic samples; **c** gene abundance related to flagellar assembly in cecal samples; **d** gene abundance related to flagellar assembly in colonic samples. Values are presented as the mean of hit counts per dietary starch. Hit counts were normalized with DESeq2-size factors. Only the taxonomic identity of the 10 most abundant genera is presented. Abundance change significance (*p* < 0.05 (false discovery rate, *q* < 0.10)) between dietary starches is presented in Table S7 and S8

Of note, the KO level 3 pathway ‘lipopolysaccharide biosynthesis’ was among the 10 most enriched pathways by the TGS diet at both gut sites (Fig. 4a and b; Additional file 1: Tables S1 and S2). These reads were again mostly contributed by the TGS-related increase in *Prevotella, Acidaminococcus*, *Veillonella* and *Geobacter* (*p* < 0.05) and replaced the contribution of various *Proteobacteria* related to the CON diet in both cecal and colonic samples (Fig. 4a and b Additional file 1: Table S7). Other functions related to potential virulence factors, such as genes mapping for ‘flagellar assembly’ were less frequent in TGS- compared to CON-fed pigs which could be associated with the reduced cecal and colonic abundance of *Treponema*, *Aeromonas*, *Shewanella* and *Escherichia* in TGS- compared to CON-fed pigs (Fig. 4c and d; Additional file 1: Table S8).

## Discussion

While replacing rapidly digestible starch in the diet by RS may be a healthy option to control blood sugar and to increase the daily dietary fiber intake [3, 13], this commonly affects the gut microbial community with functional adaptations in key metabolic attributes which are indicated by alterations in metabolite profiles and microbial signaling at the host mucosa [8, 9, 13]. Previously, we studied the response of the gastrointestinal microbiome to RS type 4 (i.e. enzymatically modified starch (EMS) and TGS) based on 16S rRNA gene sequencing [8, 9] which provided valuable information on RS-related alterations in diversity and taxonomic composition and allowed us to predict the cecal functional metagenome content for the EMS from the 16S rRNA dataset. However, due to the inherent limitation that 16S rRNA gene sequencing alone is insufficient to reveal microbial function, we used a whole genome shotgun metagenomics approach to characterize the bacterial functional adaptations that were associated with the taxonomic shifts caused by the TGS in the large intestine. The present shotgun metagenomic data demonstrate that replacing part of the dietary starch by TGS produced drastic and similarly directed shifts in taxonomy and functions in the cecal and colonic microbiota of growing pigs, despite the continuing changes in substrate availability from cecum to colon. In providing a deeper insight into the archaeal community than our previous 16S rRNA gene approach, results showed that TGS-related changes were mostly within *Eubacteria*, whereas only subtle changes in the archaeal community (i.e. *Crenarchaeota*) were detected. Since methanogens lack the enzymatic capacities to break down external glycans to monosaccharides, they rely on the provision of monosaccharides and hydrogen generated during the fermentation of carbohydrates [14, 15]. Consequently, the TGS-related reduced enzymatic capacities annotated in methane metabolism may indicate a lower substrate availability for methanogens in the hindgut of TGS- compared to CON-fed pigs.

In considering the nature of the alpha- and beta-glycosidic bonds in the TGS product, many of those linkages can be found in non-digestible oligo- and polysaccharides of plant, yeast and algae origin, privileging bacteria that are commonly associated with the breakdown of such oligo- and polysaccharides. While being a metabolically extremely versatile bacterial group [16], this likely explains the great contribution of *Prevotella* to the enrichment in genes for key catabolic steps. Similarly, *Bacteroides* are adept at using both complex plant and host-derived glycans as the main sources of energy [17, 18], explaining their contribution to taxonomic hit counts and metagenome functions. TGS-related alterations in digesta viscosity, intestinal substrate flow and retention in the various intestinal segments are other possible modes of action how the TGS diet may have modified the enzymatic capacities in cecal and colonic digesta. An increased flow of dietary protein and peptides in TGS-fed pigs may have stimulated bacterial amino acid metabolism in the cecal and colonic regions which would be supported by the many related genes that were enriched with the TGS compared to the CON diet. Alternatively, as sufficient energy needs to be available [19], the greater flow of starch with the TGS diet to the cecum-colon region may have stimulated bacterial amino acid synthesis, leading to the enrichment in the respective pathways. Against this background, the drastic increase in *Acidaminococcus*, for instance, may be more related to greater substrate flow to the cecum and colon and availability of amino acids as their main carbon source (e.g. glutamate) [20] in TGS-fed pigs than to the TGS itself. Other taxa, such as *Eubacterium* and *Veillonella*, may have relied on cross-feeding of primary fermentation metabolites such as lactate and succinate for the formation of propionate and butyrate, respectively [21]. This clearly implies that the different metabolic pathways used for the breakdown of the two dietary starches resulted in the production of different primary metabolites which then modulated the abundance of taxa relying on them. This assumption is supported by the increase in succinyl-CoA synthetase [22] and decrease in acetate and butyrate kinases [21] as well as by our findings for enhanced cecal and colonic lactate and propionate concentrations with the TGS diet, respectively [9].

The NMDS ordination plots showed strong differences in the predicted bacterial functions, supporting that the TGS required a different set of alpha- and beta-glucanases to deconstruct the additional linkages produced during transglycosylation compared to the waxy cornstarch. Although the TGS diet only decreased the hit count abundance of the KEGG pathway ‘starch and sucrose metabolism’ by about 0.25 log_2_ fold in both cecal and colonic samples, the catabolic abilities within this pathway as well as the origin of those genes largely differed. Since the taxonomic and functional prediction is based on the alignment of short 150 bp fragments, care should be taken in the interpretation of the obtained origin of the predicted functions. The decline in the predicted alpha-amylases and maltases was assignable to the decrease in *Bacillus*, *Aeromonas*, *Streptococcus* and *Clostridium* with the TGS compared to the CON diet. Enzymes within the KEGG pathways ‘galactose metabolism’ and ‘fructose and mannose metabolism’ may have been useful in the breakdown of TGS, especially enzymes predicted to encode beta-galactosidases. This enzyme capacity is involved in the hydrolysis of beta-glycosidic bonds in beta-galactosides, including lactose [11, 23], and has been reported to decrease in the intestinal metagenome of pigs after weaning due to the weaning-related decrease in *Bacteroides* and increase in *Prevotella* [24]. Compared to the weaning shift, the predicted three-time greater contribution of *Prevotella* to the beta-galactosidase genes indicated a growth advantage for this genus in pigs fed the TGS diet compared to *Bacteroides*. Moreover, two scenarios may explain the enrichment of the TGS-metagenomes with alpha-galactosidase (*galA*), with the effect being stronger in colonic than in cecal samples. Alpha-galactosidase (*galA*) capacities are involved in the degradation of glycoproteins such as mucins [25], which may have played a greater role as bacterial substrate in the colon compared to the cecum in TGS-fed pigs. Alternatively, bacterial alpha-galactosidase activity breaks down melibiose [26], the chiral form of lactose. Therefore, the enhanced predicted alpha-galactosidase activity in colonic digesta of TGS-fed pigs may have been also in relation to a greater flow of slowly fermentable TGS fragments to the colonic region. In line with this assumption, the TGS-associated enrichment in the predicted KEGG pathway ‘citrate cycle’ supports enhanced fermentative activity and bacterial energy acquisition in the cecum and colon of TGS- compared to CON-fed pigs. Moreover, the metabolic pathways feeding intermediates into this pathway differed between diets. In CON-fed pigs, glycolysis and pyruvate metabolism probably provided the necessary precursors [15], whereas in TGS-fed pigs the predicted ‘pentose and glucuronate interconversions’ pathway may have played a greater role in transferring metabolites from the TGS-enriched KEGG pathways ‘galactose metabolism’ and ‘fructose and mannose metabolism’ to provide precursors for the ‘citrate cycle’, ‘amino sugar and nucleotide sugar metabolism’ [15], thereby stimulating the biosynthesis of B-vitamins and ascorbate. Despite the less efficient absorption of vitamins from the large intestinal segments, an enhanced bacterial vitamin synthesis may still benefit the host animal [27, 28]. While those predicted genes were enriched, predicted DNA replication, transcription and translation processes were repressed in cecal and colonic metagenomes of TGS- compared to CON-fed pigs, potentially indicating bacterial metabolic priorities at the time point of sampling.

Besides utilizing diet- and host-related substrates, the intestinal microbiota serve as a rich source of immune-reactive molecules [29, 30]. Due to the taxonomic enrichment with Gram-negative bacteria, especially *Prevotella*, *Acidaminococcus,* and *Veillonella*, the cecal and colonic metagenomes of TGS-fed pigs comprised more reads predicted to encode proteins for the biosynthesis of lipopolysaccharides; a potent stimulant of the mucosal innate immune response by binding to Toll-like receptor 4 (TLR4) [31]. As the fatty acid composition of the lipid A component of lipopolysaccharides is responsible for the activation of the TLR4 response and differs among the various Gram-negative bacteria [32], the decrease in the *TLR4* expression that we observed for the cecal and colonic mucosa [11] may be directly related to the taxonomic identity of those reads as well as to the development of a certain immune tolerance [30]. For instance, the predicted contribution of *Prevotella* to the lipopolysaccharides biosynthesis genes increased from about 50% in CON-fed pigs to more than 80% in TGS-fed pigs, lowering the predicted contribution of *Proteobacteria* whose lipopolysaccharides commonly provoke a strong immune response [33]. With the associated decrease in *Proteobacteria*, e.g. *Aeromonas* and *Vibrio*, the metagenomes of TGS-fed pigs also became depleted with other genes predicted to code for potential virulence factors, such as ‘flagellar assembly’, which play a significant role in enhancing the pathogen’s capability to cause disease [34]. This may have contributed to the lower expression of genes related to the innate immune response in TGS-fed pigs as well, which we reported in our companion article [11]. These findings for the TGS show the importance of the provenience of microbial immune stimulants and emphasizes the necessity to discover their bacterial origin in relation to dietary changes in human and animal studies.

## Conclusion

The present shotgun metagenomics approach demonstrated that replacing part of the dietary starch by TGS produced similarly taxonomic and functional shifts in the microbial community with some variations due to substrate availability in the cecum-colon region of growing pigs. Samples showed strong discrimination to the respective starch in pig’s diet by altered functional capacities for amino acid, carbohydrate, and energy metabolism, synthesis of vitamins and virulence factors, as well as cellular information processing. Key enzymatic capacities for the breakdown of TGS may be found within the KO pathways ‘galactose metabolism’ and ‘fructose and mannose metabolism’. Present results also emphasize that changes in the abundance and provenience of microbial immune stimulants necessitate to discover their bacterial origin in relation to dietary changes and to assess potential consequences for host physiology, metabolism and health. Nevertheless, it needs to be considered that the present shotgun metagenomic approach based on multiplexing of 150-bp DNA libraries can only provide a prediction of the bacterial metabolic capacities and should be complemented by metatranscriptomics and metaproteomics in the future.

## Methods

### Animal experiment

The experimental design and diets of this study have been described in the sister article [9], presenting data from the same pigs as used in this study. In brief, 16 growing male Large White pigs (age: 4 months; initial BW: 45.4 ± 4.2 kg) from 6 litters were obtained from the University research farm (University of Veterinary Medicine Vienna, Vienna, Austria) and randomly assigned to 1 of 2 diets in a randomized design with 2 replicate batches, with 4 animals per treatment in each batch [9]. Pigs were individually fed and housed in metabolism pens (1.20 m × 1.00 m) with Plexiglas walls to allow visual contact. Each cage was equipped with one nipple drinker with free access to demineralized water, one feeder and one heating lamp.

### Diets and feeding

The two semi-purified diets consisted of purified cornstarch, casein, lignocellulose (FibreCell M1; agromed Austria GmbH, Kremsmünster, Austria), rapeseed oil, vitamins, and minerals and were identical except for the starch component [9] and were formulated to meet or exceed current recommendations for nutrient requirements for growing pigs [35]. The CON diet comprised a rapidly digestible waxy cornstarch (Agrana Research and Innovation Center GmbH (ARIC), Tulln, Austria), whereas 50% of the native waxy cornstarch was replaced by the TGS product (ARIC) in the TGS diet [9]. The TGS was produced via an acid-catalyzed transglycosylation of the native waxy cornstarch, which rearranges the glycosidic bonds. As a result, the TGS had 8 types of glycosydic bonds (i.e. α(1,2), α(1,3), α(1,4), α(1,6), β(1,2), β(1,3), β(1,4), and β(1,6)-glycosidic bonds) and a total dietary fiber content of 50% (method 2009.01) [36], whereas the native waxy cornstarch had only α(1,4)- and α(1,6)-glycosidic bonds.

Pigs were manually fed the experimental diets three times daily (0800, 1100 and 1600 h) to mimic human meal patterns, whereby feed allowances were calculated to surpass pig’s appetite [9]. Feed spillage and residuals in feeding bowls were collected after feeding. Diets were analyzed for dry matter, crude protein, calcium, phosphorus and starch as previously described [9, 11].

### Collection of intestinal digesta samples

Intestinal digesta samples were collected 2 h after morning feeding. After sedation (Narketan, 10 ml/kg body weight; Ketamine HCl; Vétoquinol AG, Ittigen, Austria; and Stresnil, 3 ml/kg body weight; Azaperone; Biokema SA, Crissier, Switzerland), pigs were euthanized by intracardiac injection of T61 (10 ml/kg, Embutramide; MSD Animal Health, Vienna, Austria) [9]. The abdominal cavity was opened. The whole gastrointestinal tract was removed from the abdomen, and the small and large intestines were identified, isolated, and carefully dissected them from the mesentery. The cecum and mid colon (top of the beehive) were identified, opened at the mesentery and emptied. The luminal digesta was collected, thoroughly homogenized and stored on ice until long-term storage at − 80 °C.

### DNA isolation and shotgun metagenomic sequencing

Total DNA was isolated from approximately 250 mg of cecal, and mid-colonic digesta using the PowerSoil DNA isolation kit (MoBio Laboratories, Carlsbad, CA) according to the manufacturer’s instructions except an additional heating step at 70 °C for 10 min between mixing the digesta samples with C1 buffer and bead beating to ensure proper lysis of bacteria [9, 37]. The DNA concentration was measured with a Qubit 2.0 fluorometer (Life Technologies, Carlsbad, CA) using the Qubit double-stranded DNA HS assay kit (Life Technologies, Carlsbad, CA). DNA isolates were sent to Microsynth (Balgach, Switzerland) for shotgun metagenome sequencing using the Illumina NextSeq 500 v2 sequencing platform (Illumina Inc., San Diego, CA).Total genomic DNA was prepared using the Illumina TruSeq Reagent Chemistry and the Illumina TruSeq nano protocol for whole genome shotgun sequencing of multiplexed 150-bp libraries by Microsynth AG (Balgach, Switzerland) using a high-output, single-end protocol. Barcoded sample libraries were sequenced and FASTQ files were de-multiplexed, quality filtered, and trimmed of Illumina adaptor residuals to 150 bp by Microsynth AG, yielding an average of 12–17 million reads per sample.

### Bioinformatic processing of sequences

The taxonomic and functional profiles were built by processing and annotating the quality-filtered DNA sequences using the MG-RAST (Metagenomics Rapid Annotation using Subsystem Technology, v4.0; Agronne National Laboratories; https://metagenomics.anl.gov/) pipeline [38–40]. After removal of host genomic DNA, duplicate reads and 16S rRNA reads, putative protein coding features were predicted using GragGeneScan [41] and clustered at 90% identity. Protein similarity search against the M5NR protein database was done with sBLAT [42]. Cecal and colonic samples from the same pig were treated as separate samples and processed independently. The Kyoto Encyclopedia of Genes and Genomes (KEGG) [43, 44] and KEGG orthology (KO) annotation source of MG-RAST were used for taxonomic and functional analysis of cecal and colonic samples. Subsets of the reads annotated in a KO class for taxonomic identification were created in MG-RAST. The criteria applied for inclusion were a maximum e-value cutoff of 1e-05, a minimum identity of 60% and a minimum alignment length of 15.

### Statistical analysis

A power test analysis estimated as described in Metzler-Zebeli et al. [11] based on recent data for intestinal microbiome composition [9] using the SAS software (version 9.4; SAS Inst. Inc., Cary, NC, USA) was performed to identify the number of observations needed for the present pig experiment. The power test analysis indicated that a statistical power of more than 90% for a sample size of *n* = 8 and α = 0.05 could be expected, enabling sufficient power to reject the null-hypothesis (H0), if H0 was false (*p* = 1-β).

The “DESeq” function within the DESeq2 package (version 1.14.1) [45] in R was used to test for differentially abundant taxa and putative functions by dietary starch, at each taxonomic and functional level. Data were normalized to the size factors of the libraries and dispersion estimation and were listed as normalized read counts per feature. This function models raw counts using a negative binomial distribution and adjusts internally for “size factors” which normalize for differences in sequencing depth between sample libraries. In addition, we pre-filtered the taxonomic and functional datasets to keep only features that have at least 10 reads total using the R command in DESeq2 “rowSums(counts(deseq_data)) ≥ 10” to remove low-count taxa and functions for the phylum through species level analysis as well as KO level 3 and KO function analysis.

DEseq2 default settings were used to replace and filter for count outliers. Differential taxa and function abundance between treatments were identified using the “Wald” test [45]. Data were listed as normalized read counts per feature. The correction of *p* values relating to the taxonomic and functional profiles were performed using the Benjamini-Hochberg false discovery rate (FDR) [46]. To account for the multiple comparisons at each taxonomic and functional level, we considered a type I error rate of ≤0.05 and a FDR-adjusted *p* value (*q* value) ≤ 0.10 as significant. Mean counts for each dietary starch were computed using the “sapply” function in DESeq2. Log-transformed DESeq2-normalized data were used to create heatmaps and to compute distance matrices (Euclidian distance) for bacterial phyla and genera and functional attributes (KO level 3) using the pheatmap R package in R. Statistical assessment of dissimilarity matrices (Bray-Curtis) from the functional data was done using NMDS with the “metaMDS” function in the vegan R package (version 2.5.1) [47]. All statistical tests were carried out using R studio (version 1.0.136).

## Additional file

Additional file 1: **Table S1.** Selected KEGG orthology functions within the KEGG pathway ‘carbohydrate metabolism’ in cecal samples being differently enriched in CON- and TGS-fed pigs. **Table S2.** Selected KEGG orthology functions within the KEGG pathway ‘carbohydrate metabolism’ in colonic samples being differently enriched in CON- and TGS-fed pigs. **Table S3.** Taxonomic identity of *lacZ* genes being differently enriched in cecal and colonic samples of CON- and TGS-fed pigs. **Table S4.** Taxonomic identity of *galA* genes being differently enriched in cecal and colonic samples of CON- and TGS-fed pigs. **Table S5.** Taxonomic identity of *amyA* genes being differently enriched in cecal and colonic samples of CON- and TGS-fed pigs. **Table S6.** Taxonomic identity of *araA* genes being differently enriched in cecal and colonic samples of CON- and TGS-fed pigs. **Table S7.** Taxonomic identity of genes predicted to encode proteins within the KEGG pathway ‘lipopolysaccharide biosynthesis’ differently enriched in cecal and colonic samples of CON- and TGS-fed pigs. **Table S8.** Taxonomic identity of genes predicted to encode proteins within the KEGG pathway ‘flagellar assembly’ differently enriched in cecal and colonic samples of CON- and TGS-fed pigs. **Figure S1.** Heatmaps of abundances (hit counts) and distance matrices (Euclidian distance) of cecal and colonic samples at phylum level in control starch (CON)- and transglycosylated starch (TGS)-fed pigs (P1-P16). a phyla abundances in cecum; b phyla abundances in colon; c distance matrix of cecal samples; and d distance matrix of colonic samples. Hit counts were normalized with DESeq2-size factors. Abundance change significance between dietary starches is indicated by * (*p* < 0.05 (false discovery rate, *q* < 0.10)). **Figure S2.** Heatmaps of differently abundant genera (hit counts) in of cecal (a) and colonic samples (b) from control starch (CON)- and transglycosylated starch (TGS)-fed pigs (P1-P16). Only the 30 most abundant genera that were differently affected by the dietary starch source are presented. Hit counts were normalized with DESeq2-size factors. Abundance change significance between dietary starches is *p* < 0.05 (false discovery rate, *q* < 0.10)). (PDF 286 kb) (PDF 250 kb)

## Abbreviations

CON: Control starch
KEGG: Kyoto Encyclopedia of Genes and Genomes
KO: KEGG orthology
MG-RAST: Metagenomics Rapid Annotation using Subsystem Technology
NMDS: Non-parametric multidimensional scaling
RS: Resistant starch
SCFA: Short-chain fatty acids
TGS: Transglycosylated starch
