# Supplementary material for: Functional adaptations in the cecal and colonic metagenomes associated with the consumption of transglycosylated starch in a pig model
Source: BMC Microbiol. 2019 May 2;19:87. doi: 10.1186/s12866-019-1462-2 (PMC6498482; doi:10.1186/s12866-019-1462-2)
Supplement: Supplementary file 1 — Table S1. Selected KEGG orthology functions within the KEGG pathway ‘carbohydrate metabolism’ in cecal samples being differently enriched in CON- and TGS-fed pigs. Table S2. Selected KEGG orthology functions within the KEGG pathway ‘carbohydrate metabolism’ in colonic samples being differently enriched in CON- and TGS-fed pigs. Table S3. Taxonomic identity of lacZ genes being differently enriched in cecal and colonic samples of CON- and TGS-fed pigs. Table S4. Taxonomic identity of galA genes being differently enriched in cecal and colonic samples of CON- and TGS-fed pigs. Table S5. Taxonomic identity of amyA genes being differently enriched in cecal and colonic samples of CON- and TGS-fed pigs. Table S6. Taxonomic identity of araA genes being differently enriched in cecal and colonic samples of CON- and TGS-fed pigs. Table S7. Taxonomic identity of genes predicted to encode proteins within the KEGG pathway ‘lipopolysaccharide biosynthesis’ differently enriched in cecal and colonic samples of CON- and TGS-fed pigs. Table S8. Taxonomic identity of genes predicted to encode proteins within the KEGG pathway ‘flagellar assembly’ differently enriched in cecal and colonic samples of CON- and TGS-fed pigs. Figure S1. Heatmaps of abundances (hit counts) and distance matrices (Euclidian distance) of cecal and colonic samples at phylum level in control starch (CON)- and transglycosylated starch (TGS)-fed pigs (P1-P16). a phyla abundances in cecum; b phyla abundances in colon; c distance matrix of cecal samples; and d distance matrix of colonic samples. Hit counts were normalized with DESeq2-size factors. Abundance change significance between dietary starches is indicated by * (p < 0.05 (false discovery rate, q < 0.10)). Figure S2. Heatmaps of differently abundant genera (hit counts) in of cecal (a) and colonic samples (b) from control starch (CON)- and transglycosylated starch (TGS)-fed pigs (P1-P16). Only the 30 most abundant genera that were differently affected by the diet [file 12866_2019_1462_MOESM1_ESM.pdf]

Functional adaptations in the cecal and colonic metagenomes associated with the consumption of transglycosylated starch in a pig model

Barbara U. Metzler-Zebeli[1], Monica A. Newman[1], Dietmar Grüll[2], Qendrim Zebeli[1]

[1]Institute of Animal Nutrition and Functional Plant Compounds, Department for Farm Animals and Veterinary Public Health, University of Veterinary Medicine Vienna, 1210 Vienna, Austria

[2]Agrana Research & Innovation Center GmbH, 3430 Tulln, Austria;

Short title: Resistant starch and functional metagenome

Corresponding author: Barbara U. Metzler-Zebeli. E-mail: barbara.metzler@vetmeduni.ac.at

Table S1 Selected KEGG orthology functions within the KEGG pathway 'carbohydrate metabolism' in cecal samples being differently enriched in CON- and TGS-fed pigs

KEGG orthology function	Mean*	$\log_2$ fold change	SE†	*p* value	*q* value‡
E3.2.1.85 lacG; 6-phospho-beta-galactosidase [EC:3.2.1.85]	274	2.945	0.622	<0.001	<0.001
uxuB; fructuronate reductase [EC:1.1.1.57]	1015	2.576	0.351	<0.001	<0.001
araA; L-arabinose isomerase [EC:5.3.1.4]	1315	1.969	0.348	<0.001	<0.001
gatY-kbaY; tagatose 16-diphosphate aldolase GatY/KbaY [EC:4.1.2.40]	130	1.892	0.350	<0.001	<0.001
E3.1.2.1 ACH1; acetyl-CoA hydrolase [EC:3.1.2.1]	491	1.764	0.324	<0.001	<0.001
E3.2.1.86B bglA; 6-phospho-beta-glucosidase [EC:3.2.1.86]	653	1.442	0.355	<0.001	<0.001
korD; 2-oxoglutarate ferredoxin oxidoreductase subunit delta [EC:1.2.7.3]	146	1.421	0.273	<0.001	<0.001
E3.2.1.22B galA rafA; alpha-galactosidase [EC:3.2.1.22]	3262	1.393	0.255	<0.001	<0.001
fucK; L-fuculokinase [EC:2.7.1.51]	213	1.348	0.294	<0.001	<0.001
E4.1.1.70; glutaconyl-CoA decarboxylase [EC:4.1.1.70]	504	1.321	0.228	<0.001	<0.001
E3.2.1.122 glvA; maltose-6'-phosphate glucosidase [EC:3.2.1.122]	107	1.311	0.303	<0.001	<0.001
E3.2.1.1A; alpha-amylase [EC:3.2.1.1]	1281	1.256	0.244	<0.001	<0.001
E2.7.1.4 scrK; fructokinase [EC:2.7.1.4]	1882	1.245	0.231	<0.001	<0.001
nagB GNPDA; glucosamine-6-phosphate deaminase [EC:3.5.99.6]	2370	1.233	0.225	<0.001	<0.001
sdhC; succinate dehydrogenase cytochrome b556 subunit	468	1.222	0.264	<0.001	<0.001
murQ; N-acetylmuramic acid 6-phosphate etherase [EC:4.2.1.126]	595	1.195	0.203	<0.001	<0.001
korG; 2-oxoglutarate ferredoxin oxidoreductase subunit gamma [EC:1.2.7.3]	623	1.112	0.263	<0.001	<0.001
lacZ; beta-galactosidase [EC:3.2.1.23]	6502	1.047	0.235	<0.001	<0.001
sdhB; succinate dehydrogenase iron-sulfur subunit [EC:1.3.99.1]	967	1.028	0.227	<0.001	<0.001
uxuA; mannonate dehydratase [EC:4.2.1.8]	1029	1.006	0.228	<0.001	<0.001
uxaC; glucuronate isomerase [EC:5.3.1.12]	1147	0.959	0.222	<0.001	<0.001
GPI pgi; glucose-6-phosphate isomerase [EC:5.3.1.9]	3088	-0.859	0.201	<0.001	<0.001
PRPS prsA; ribose-phosphate pyrophosphokinase [EC:2.7.6.1]	1899	-1.018	0.199	<0.001	<0.001
TPI tpiA; triosephosphate isomerase (TIM) [EC:5.3.1.1]	1394	-1.186	0.270	<0.001	<0.001
glmM; phosphoglucosamine mutase [EC:5.4.2.10]	1309	-1.272	0.289	<0.001	<0.001
xylA; xylose isomerase [EC:5.3.1.5]	233	-1.374	0.314	<0.001	<0.001
glgC; glucose-1-phosphate adenylyltransferase [EC:2.7.7.27]	1697	-1.567	0.223	<0.001	<0.001
accC; acetyl-CoA carboxylase biotin carboxylase subunit [EC:6.4.1.2 6.3.4.14]	913	-1.655	0.372	<0.001	<0.001
E1.4.7.1; glutamate synthase (ferredoxin) [EC:1.4.7.1]	942	-1.996	0.419	<0.001	<0.001
accB bccP; acetyl-CoA carboxylase biotin carboxyl carrier protein	158	-2.032	0.400	<0.001	<0.001
ldhA; D-lactate dehydrogenase [EC:1.1.1.28]	615	-2.193	0.421	<0.001	<0.001
E3.2.1.1 amyA malS; alpha-amylase [EC:3.2.1.1]	1038	-2.257	0.542	<0.001	<0.001
rpiA; ribose 5-phosphate isomerase A [EC:5.3.1.6]	261	-2.832	0.639	<0.001	<0.001
purU; formyltetrahydrofolate deformylase [EC:3.5.1.10]	195	-3.128	0.563	<0.001	<0.001
pta; phosphate acetyltransferase [EC:2.3.1.8]	258	-3.240	0.644	<0.001	<0.001
acnB; aconitate hydratase 2 / 2-methylisocitrate dehydratase [EC:4.2.1.3 4.2.1.99]	472	-3.434	0.625	<0.001	<0.001
ppgK; polyphosphate glucokinase [EC:2.7.1.63]	115	-3.943	0.888	<0.001	<0.001
xynB; xylan 14-beta-xylosidase [EC:3.2.1.37]	236	1.803	0.463	<0.001	0.001

treC; trehalose-6-phosphate hydrolase [EC:3.2.1.93]	236	1.078	0.282	<0.001	0.001
fbp3; fructose-16-bisphosphatase III [EC:3.1.3.11]	1281	0.763	0.201	<0.001	0.001
E5.1.3.3 galM; aldose 1-epimerase [EC:5.1.3.3]	1481	0.742	0.199	<0.001	0.001
E5.3.1.8 manA; mannose-6-phosphate isomerase [EC:5.3.1.8]	1059	0.690	0.189	<0.001	0.001
E2.4.1.21 glgA; starch synthase [EC:2.4.1.21]	1490	-0.590	0.161	<0.001	0.001
E2.4.1.1 glgP PYG; starch phosphorylase [EC:2.4.1.1]	4517	-0.647	0.172	<0.001	0.001
pfkA PFK; 6-phosphofructokinase 1 [EC:2.7.1.11]	2368	-0.906	0.242	<0.001	0.001
E2.5.1.56 neuB; N-acetylneuraminate synthase [EC:2.5.1.56]	707	-1.100	0.300	<0.001	0.001
IDH3; isocitrate dehydrogenase (NAD+) [EC:1.1.1.41]	179	-1.451	0.395	<0.001	0.001
accA; acetyl-CoA carboxylase carboxyl transferase subunit alpha [EC:6.4.1.2]	524	-2.003	0.517	<0.001	0.001
E2.7.1.17; xylulokinase [EC:2.7.1.17]	1305	0.991	0.275	<0.001	0.002
accD; acetyl-CoA carboxylase carboxyl transferase subunit beta [EC:6.4.1.2]	449	-1.849	0.511	<0.001	0.002
manC cpsB; mannose-1-phosphate guanylyltransferase [EC:2.7.7.13]	1845	0.990	0.293	0.001	0.003
araD; L-ribulose-5-phosphate 4-epimerase [EC:5.1.3.4]	407	0.893	0.260	0.001	0.003
FBA fbaA; fructose-bisphosphate aldolase class II [EC:4.1.2.13]	2405	-0.715	0.208	0.001	0.003
mtlD; mannitol-1-phosphate 5-dehydrogenase [EC:1.1.1.17]	170	1.487	0.449	0.001	0.004
UGP2 galU galF; UTP--glucose-1-phosphate uridylyltransferase [EC:2.7.7.9]	731	-0.784	0.235	0.001	0.004
E2.2.1.1 tktA tktB; transketolase [EC:2.2.1.1]	4254	-0.631	0.200	0.002	0.007
E4.2.1.47 gmd; GDPmannose 46-dehydratase [EC:4.2.1.47]	1773	0.643	0.214	0.003	0.011
CS gltA; citrate synthase [EC:2.3.3.1]	2121	-0.493	0.165	0.003	0.011
E6.4.1.1B pycB; pyruvate carboxylase subunit B [EC:6.4.1.1]	1294	0.425	0.144	0.003	0.012
korB; 2-oxoglutarate ferredoxin oxidoreductase subunit beta [EC:1.2.7.3]	1244	0.477	0.164	0.004	0.014
deoC DERA; deoxyribose-phosphate aldolase [EC:4.1.2.4]	1639	0.504	0.175	0.004	0.015
glpX; fructose-16-bisphosphatase II [EC:3.1.3.11]	328	-1.372	0.485	0.005	0.017
E3.1.1.11; pectinesterase [EC:3.1.1.11]	326	1.078	0.384	0.005	0.018
murA; UDP-N-acetylglucosamine 1-carboxyvinyltransferase [EC:2.5.1.7]	3145	-0.848	0.303	0.005	0.018
glcD; glycolate oxidase [EC:1.1.3.15]	534	0.843	0.305	0.006	0.019
PC pyc; pyruvate carboxylase [EC:6.4.1.1]	370	0.460	0.166	0.006	0.019
E3.2.1.52 nagZ; beta-N-acetylhexosaminidase [EC:3.2.1.52]	442	-0.935	0.342	0.006	0.021
RENBP; N-acylglucosamine 2-epimerase [EC:5.1.3.8]	489	0.566	0.211	0.007	0.024
E2.7.1.90 pfk; pyrophosphate--fructose-6-phosphate 1-phosphotransferase [EC:2.7.1.90]	1705	0.393	0.149	0.008	0.026
mdh; malate dehydrogenase [EC:1.1.1.37]	1298	0.683	0.265	0.010	0.031
wbpO; UDP-N-acetyl-D-galactosamine dehydrogenase [EC:1.1.1.-]	849	0.649	0.252	0.010	0.031
E3.2.1.55 abfA; alpha-N-arabinofuranosidase [EC:3.2.1.55]	823	0.836	0.330	0.011	0.033
pps ppsA; pyruvate water dikinase [EC:2.7.9.2]	301	0.482	0.189	0.011	0.033
rpe RPE; ribulose-phosphate 3-epimerase [EC:5.1.3.1]	1417	-0.401	0.160	0.012	0.035
ackA; acetate kinase [EC:2.7.2.1]	2673	-0.928	0.373	0.013	0.036
lacC; tagatose 6-phosphate kinase [EC:2.7.1.144]	126	0.864	0.350	0.014	0.038
E1.1.1.271 fcl; GDP-L-fucose synthase [EC:1.1.1.271]	1464	0.519	0.217	0.017	0.046
korA; 2-oxoglutarate ferredoxin oxidoreductase subunit alpha [EC:1.2.7.3]	1830	0.390	0.164	0.018	0.048
E3.2.1.10; oligo-16-glucosidase [EC:3.2.1.10]	365	0.523	0.226	0.021	0.055
bcsA; cellulose synthase (UDP-forming) [EC:2.4.1.12]	90	-1.517	0.659	0.021	0.056
kdgK; 2-dehydro-3-deoxygluconokinase [EC:2.7.1.45]	1398	0.433	0.193	0.025	0.064

wecC; UDP-N-acetyl-D-mannosaminuronic acid dehydrogenase [EC:1.1.1.336]	145	1.074	0.485	0.027	0.069
glmU; bifunctional UDP-N-acetylglucosamine pyrophosphorylase / Glucosamine-1-phosphate N-acetyltransferase [EC:2.7.7.23 2.3.1.157]	779	-0.646	0.296	0.029	0.074
E1.1.1.44 PGD gnd; 6-phosphogluconate dehydrogenase [EC:1.1.1.44]	102	0.981	0.458	0.032	0.080
npdA; NAD-dependent deacetylase [EC:3.5.1.-]	1262	-0.368	0.172	0.032	0.080
E2.7.2.7 buk; butyrate kinase [EC:2.7.2.7]	424	-0.729	0.350	0.037	0.091

*Normalized reads (hit counts). Only the most abundant functions (>0.01% of all reads) that were altered by the dietary starch source are presented (*n* = 8 per diet).

† Standard error of the $\log_2$fold change.

‡ False discovery rate (Benjamini-Hochberg) corrected *p* value.

Table S2 Selected KEGG orthology functions within the KEGG pathway 'carbohydrate metabolism' in colonic samples being differently enriched in CON- and TGS-fed pigs

KEGG orthology function	Mean*	$\log_2$ fold change	SE†	*p* value	*q* value‡
E3.2.1.85 lacG; 6-phospho-beta-galactosidase [EC:3.2.1.85]	483	5.117	0.371	<0.001	<0.001
xynB; xylan 14-beta-xylosidase [EC:3.2.1.37]	221	2.411	0.374	<0.001	<0.001
gatY-kbaY; tagatose 16-diphosphate aldolase GatY/KbaY [EC:4.1.2.40]	286	2.386	0.334	<0.001	<0.001
E3.2.1.86B bglA; 6-phospho-beta-glucosidase [EC:3.2.1.86]	1097	2.316	0.331	<0.001	<0.001
E3.1.2.1 ACH1; acetyl-CoA hydrolase [EC:3.1.2.1]	544	2.243	0.167	<0.001	<0.001
ulaG; L-ascorbate 6-phosphate lactonase [EC:3.1.1.-]	147	2.139	0.379	<0.001	<0.001
uxuB; fructuronate reductase [EC:1.1.1.57]	741	1.956	0.232	<0.001	<0.001
treC; trehalose-6-phosphate hydrolase [EC:3.2.1.93]	430	1.939	0.297	<0.001	<0.001
araA; L-arabinose isomerase [EC:5.3.1.4]	1206	1.882	0.218	<0.001	<0.001
ppc; phosphoenolpyruvate carboxylase [EC:4.1.1.31]	322	1.877	0.468	0.000	<0.001
E4.1.1.70; glutaconyl-CoA decarboxylase [EC:4.1.1.70]	750	1.876	0.247	<0.001	<0.001
sdhC; succinate dehydrogenase cytochrome b556 subunit	407	1.691	0.156	<0.001	<0.001
glcD; glycolate oxidase [EC:1.1.3.15]	792	1.617	0.239	<0.001	<0.001
murQ; N-acetylmuramic acid 6-phosphate etherase [EC:4.2.1.126]	861	1.483	0.203	<0.001	<0.001
E3.2.1.122 glvA; maltose-6'-phosphate glucosidase [EC:3.2.1.122]	163	1.456	0.274	<0.001	<0.001
E3.2.1.22B galA rafA; alpha-galactosidase [EC:3.2.1.22]	3509	1.332	0.149	<0.001	<0.001
E3.2.1.1A; alpha-amylase [EC:3.2.1.1]	1049	1.260	0.158	<0.001	<0.001
manC cpsB; mannose-1-phosphate guanylyltransferase [EC:2.7.7.13]	1297	1.227	0.161	<0.001	<0.001
sdhB; succinate dehydrogenase iron-sulfur subunit [EC:1.3.99.1]	800	1.219	0.152	<0.001	<0.001
lacZ; beta-galactosidase [EC:3.2.1.23]	5813	1.165	0.134	<0.001	<0.001
korD; 2-oxoglutarate ferredoxin oxidoreductase subunit delta [EC:1.2.7.3]	160	1.151	0.161	<0.001	<0.001
araD; L-ribulose-5-phosphate 4-epimerase [EC:5.1.3.4]	476	1.109	0.215	<0.001	<0.001
E4.2.1.47 gmd; GDPmannose 46-dehydratase [EC:4.2.1.47]	1599	1.063	0.173	<0.001	<0.001
pps ppsA; pyruvate water dikinase [EC:2.7.9.2]	500	1.047	0.213	<0.001	<0.001
E2.7.1.4 scrK; fructokinase [EC:2.7.1.4]	1799	1.032	0.144	<0.001	<0.001
nagB GNPDA; glucosamine-6-phosphate deaminase [EC:3.5.99.6]	2393	1.010	0.154	<0.001	<0.001
mdh; malate dehydrogenase [EC:1.1.1.37]	1159	0.870	0.188	<0.001	<0.001
korG; 2-oxoglutarate ferredoxin oxidoreductase subunit gamma [EC:1.2.7.3]	610	0.821	0.150	<0.001	<0.001
E5.3.1.8 manA; mannose-6-phosphate isomerase [EC:5.3.1.8]	1025	0.792	0.143	<0.001	<0.001
fbp3; fructose-16-bisphosphatase III [EC:3.1.3.11]	1421	0.774	0.121	<0.001	<0.001
E3.5.1.25 nagA AMDHD2; N-acetylglucosamine-6-phosphate deacetylase [EC:3.5.1.25]	876	0.770	0.186	<0.001	<0.001
E1.1.1.271 fcl; GDP-L-fucose synthase [EC:1.1.1.271]	1226	0.702	0.180	<0.001	<0.001
deoC DERA; deoxyribose-phosphate aldolase [EC:4.1.2.4]	1573	0.607	0.158	<0.001	<0.001
korA; 2-oxoglutarate ferredoxin oxidoreductase subunit alpha [EC:1.2.7.3]	2253	0.596	0.151	<0.001	<0.001
E2.2.1.1 tktA tktB; transketolase [EC:2.2.1.1]	5033	-0.563	0.135	<0.001	<0.001
E2.4.1.1 glgP PYG; starch phosphorylase [EC:2.4.1.1]	5262	-0.651	0.133	<0.001	<0.001
PGK pgk; phosphoglycerate kinase [EC:2.7.2.3]	3715	-0.689	0.139	<0.001	<0.001
FBA fbaA; fructose-bisphosphate aldolase class II [EC:4.1.2.13]	2598	-0.724	0.111	<0.001	<0.001

ppdK; pyruvateorthophosphate dikinase [EC:2.7.9.1]	5292	-0.816	0.203	<0.001	<0.001
PRPS prsA; ribose-phosphate pyrophosphokinase [EC:2.7.6.1]	2313	-0.849	0.150	<0.001	<0.001
E1.4.7.1; glutamate synthase (ferredoxin) [EC:1.4.7.1]	782	-0.895	0.197	<0.001	<0.001
rhaD; rhamnulose-1-phosphate aldolase [EC:4.1.2.19]	401	-0.916	0.236	<0.001	<0.001
glgC; glucose-1-phosphate adenylyltransferase [EC:2.7.7.27]	2477	-1.017	0.177	<0.001	<0.001
E3.2.1.52 nagZ; beta-N-acetylhexosaminidase [EC:3.2.1.52]	421	-1.035	0.268	<0.001	<0.001
glmM; phosphoglucosamine mutase [EC:5.4.2.10]	1981	-1.055	0.183	<0.001	<0.001
GPI pgi; glucose-6-phosphate isomerase [EC:5.3.1.9]	3330	-1.073	0.138	<0.001	<0.001
pfkA PFK; 6-phosphofructokinase 1 [EC:2.7.1.11]	3010	-1.105	0.192	<0.001	<0.001
murA; UDP-N-acetylglucosamine 1-carboxyvinyltransferase [EC:2.5.1.7]	3588	-1.257	0.169	<0.001	<0.001
ldhA; D-lactate dehydrogenase [EC:1.1.1.28]	636	-1.290	0.270	<0.001	<0.001
TPI tpiA; triosephosphate isomerase (TIM) [EC:5.3.1.1]	1811	-1.321	0.151	<0.001	<0.001
ptb; phosphate butyryltransferase [EC:2.3.1.19]	165	-1.329	0.330	<0.001	<0.001
xylA; xylose isomerase [EC:5.3.1.5]	209	-1.409	0.317	<0.001	<0.001
ackA; acetate kinase [EC:2.7.2.1]	2697	-1.411	0.142	<0.001	<0.001
IDH3; isocitrate dehydrogenase (NAD+) [EC:1.1.1.41]	211	-1.428	0.252	<0.001	<0.001
porA; pyruvate ferredoxin oxidoreductase alpha subunit [EC:1.2.7.1]	336	-1.677	0.407	0.000	<0.001
porB; pyruvate ferredoxin oxidoreductase beta subunit [EC:1.2.7.1]	274	-1.678	0.414	<0.001	<0.001
E2.7.2.7 buk; butyrate kinase [EC:2.7.2.7]	425	-1.752	0.191	<0.001	<0.001
E1.1.1.60 garR; 2-hydroxy-3-oxopropionate reductase [EC:1.1.1.60]	141	-1.790	0.335	<0.001	<0.001
accC; acetyl-CoA carboxylase biotin carboxylase subunit [EC:6.4.1.2 6.3.4.14]	1077	-1.853	0.227	<0.001	<0.001
melA; alpha-galactosidase [EC:3.2.1.22]	184	-1.978	0.295	<0.001	<0.001
accB bccP; acetyl-CoA carboxylase biotin carboxyl carrier protein	204	-2.061	0.270	<0.001	<0.001
accD; acetyl-CoA carboxylase carboxyl transferase subunit beta [EC:6.4.1.2]	518	-2.212	0.313	<0.001	<0.001
accA; acetyl-CoA carboxylase carboxyl transferase subunit alpha [EC:6.4.1.2]	659	-2.522	0.261	<0.001	<0.001
purU; formyltetrahydrofolate deformylase [EC:3.5.1.10]	168	-2.553	0.537	<0.001	<0.001
E3.2.1.1 amyA malS; alpha-amylase [EC:3.2.1.1]	1222	-2.832	0.335	<0.001	<0.001
rpiA; ribose 5-phosphate isomerase A [EC:5.3.1.6]	183	-3.345	0.492	<0.001	<0.001
pta; phosphate acetyltransferase [EC:2.3.1.8]	225	-3.624	0.569	<0.001	<0.001
lacC; tagatose 6-phosphate kinase [EC:2.7.1.144]	218	1.556	0.447	0.001	0.001
rfbH; CDP-6-deoxy-D-xylo-4-hexulose-3-dehydrase	414	1.272	0.352	<0.001	0.001
E2.7.1.17; xylulokinase [EC:2.7.1.17]	1082	0.655	0.186	<0.001	0.001
rpe RPE; ribulose-phosphate 3-epimerase [EC:5.1.3.1]	1577	-0.520	0.141	<0.001	0.001
treS; maltose alpha-D-glucosyltransferase/ alpha-amylase [EC:5.4.99.16 3.2.1.1]	506	-1.396	0.380	<0.001	0.001
E3.2.1.10; oligo-16-glucosidase [EC:3.2.1.10]	737	0.887	0.258	0.001	0.002
E5.1.3.3 galM; aldose 1-epimerase [EC:5.1.3.3]	1392	0.645	0.189	0.001	0.002
E2.7.1.90 pfk; pyrophosphate--fructose-6-phosphate 1-phosphotransferase [EC:2.7.1.90]	1278	0.581	0.170	0.001	0.002
npdA; NAD-dependent deacetylase [EC:3.5.1.-]	1265	-0.608	0.179	0.001	0.002
galE GALE; UDP-glucose 4-epimerase [EC:5.1.3.2]	4069	-0.533	0.164	0.001	0.003
acnB; aconitate hydratase 2 / 2-methylisocitrate dehydratase [EC:4.2.1.3 4.2.1.99]	447	-2.096	0.638	0.001	0.003
mtlD; mannitol-1-phosphate 5-dehydrogenase [EC:1.1.1.17]	300	1.443	0.467	0.002	0.005
E3.1.1.11; pectinesterase [EC:3.1.1.11]	181	0.982	0.318	0.002	0.005
bgaB lacA; beta-galactosidase [EC:3.2.1.23]	654	0.546	0.182	0.003	0.007

korB; 2-oxoglutarate ferredoxin oxidoreductase subunit beta [EC:1.2.7.3]	1530	0.450	0.153	0.003	0.008
wecC; UDP-N-acetyl-D-mannosaminuronic acid dehydrogenase [EC:1.1.1.336]	198	1.495	0.520	0.004	0.010
CS gltA; citrate synthase [EC:2.3.3.1]	2201	-0.471	0.165	0.004	0.010
glgB; 14-alpha-glucan branching enzyme [EC:2.4.1.18]	4168	-0.486	0.169	0.004	0.010
sucC; succinyl-CoA synthetase beta subunit [EC:6.2.1.5]	773	1.545	0.547	0.005	0.011
sucD; succinyl-CoA synthetase alpha subunit [EC:6.2.1.5]	569	1.528	0.540	0.005	0.011
ACSS acs; acetyl-CoA synthetase [EC:6.2.1.1]	2020	-0.772	0.273	0.005	0.011
E2.4.1.-; [EC:2.4.1.-]	432	0.649	0.236	0.006	0.013
E2.2.1.2 talA talB; transaldolase [EC:2.2.1.2]	316	0.564	0.207	0.006	0.014
murB; UDP-N-acetylmuramate dehydrogenase [EC:1.1.1.158]	1325	-0.374	0.139	0.007	0.015
E2.3.1.54 pflD; formate C-acetyltransferase [EC:2.3.1.54]	4467	0.430	0.167	0.010	0.020
E4.2.1.2A fumA fumB; fumarate hydratase class I [EC:4.2.1.2]	1590	0.443	0.175	0.011	0.024
E1.1.1.40 maeB; malate dehydrogenase (oxaloacetate-decarboxylating)(NADP+) [EC:1.1.1.40]	1619	0.562	0.224	0.012	0.025
E5.3.1.17 kduI; 4-deoxy-L-threo-5-hexosulose-uronate ketol-isomerase [EC:5.3.1.17]	372	0.382	0.153	0.012	0.025
fucK; L-fuculokinase [EC:2.7.1.51]	144	0.808	0.325	0.013	0.026
E3.2.1.55 abfA; alpha-N-arabinofuranosidase [EC:3.2.1.55]	751	0.677	0.278	0.015	0.029
UGP2 galU galF; UTP--glucose-1-phosphate uridylyltransferase [EC:2.7.7.9]	1083	-0.536	0.232	0.021	0.038
rhaA; L-rhamnose isomerase [EC:5.3.1.14]	782	-0.606	0.263	0.021	0.039
E2.4.1.21 glgA; starch synthase [EC:2.4.1.21]	2000	-0.322	0.145	0.026	0.046
uxaC; glucuronate isomerase [EC:5.3.1.12]	1132	0.333	0.152	0.028	0.050
rbsK RBKS; ribokinase [EC:2.7.1.15]	252	-0.549	0.263	0.037	0.062
E4.2.1.2AB fumB; fumarate hydratase subunit beta [EC:4.2.1.2]	649	-0.695	0.335	0.038	0.064
E3.2.1.20 malZ; alpha-glucosidase [EC:3.2.1.20]	2328	-0.518	0.271	0.056	0.089
E3.2.1.26 sacA; beta-fructofuranosidase [EC:3.2.1.26]	460	0.555	0.292	0.057	0.091
RENBP; N-acylglucosamine 2-epimerase [EC:5.1.3.8]	540	0.510	0.275	0.064	0.100
UGDH ugd; UDPglucose 6-dehydrogenase [EC:1.1.1.22]	2852	0.234	0.126	0.064	0.100

*Normalized reads (hit counts). Only the most abundant functions (>0.01% of all reads) that were altered by the dietary starch source are presented (*n* = 8 per diet).

† Standard error of the $\log_2$fold change.

‡ False discovery rate (Benjamini-Hochberg) corrected *p* value.

Table S3 Taxonomic identity of *lacZ* genes being differently enriched in cecal and colonic samples of CON- and TGS-fed pigs

Taxonomic identity	Mean*	log_2 fold change	SE[†]	*p* value	*q* value[‡]
Cecum					
Bacteroides	2030	0.409	0.196	0.037	0.097
Prevotella	1921	1.465	0.424	0.001	0.007
Parabacteroides	387	-0.772	0.278	0.005	0.029
Flavobacterium	92	-1.021	0.451	0.024	0.076
Paludibacter	70	-1.924	0.611	0.002	0.012
Akkermansia	62	-1.348	0.420	0.001	0.010
Eubacterium	44	0.835	0.337	0.013	0.054
Colon					
Prevotella	1577	1.897	0.316	<0.001	<0.001
Bacteroides	1556	0.529	0.215	0.014	0.021
Parabacteroides	423	-1.055	0.316	0.001	0.002
Flavobacterium	98	-1.115	0.332	0.001	0.002
Bacillus	80	1.065	0.234	<0.001	<0.001
Eubacterium	80	1.536	0.275	<0.001	<0.001
Paludibacter	66	-3.248	0.361	<0.001	<0.001
Lactobacillus	52	0.475	0.258	0.066	0.089
Butyrivibrio	44	2.377	0.330	<0.001	<0.001

*Normalized reads (hit counts). Only the most abundant genera (>0.01% of all reads) that were altered by the dietary starch source are presented ($n = 8$ per diet and gut site).

[†] Standard error of the log_2fold change.

[‡] False discovery rate (Benjamini-Hochberg) corrected *p* value.

Table S4 Taxonomic identity of *galA* genes being differently enriched in cecal and colonic samples of CON- and TGS-fed pigs

Taxonomic identity	Mean*	$\log_2$ fold change	SE[†]	*p* value	*q* value[‡]
Cecum					
Clostridium	111	-0.984	0.382	0.010	0.051
Ruminococcus	20	-1.876	0.743	0.012	0.051
Caulobacter	17	1.599	0.493	0.001	0.017
Vibrio	14	-3.823	1.457	0.009	0.051
Butyrivibrio	13	-1.534	0.594	0.010	0.051
Colon					
Parabacteroides	749	1.208	0.306	<0.001	<0.001
Bacteroides	719	1.915	0.294	<0.001	<0.001
Prevotella	429	1.944	0.362	<0.001	<0.001
Lactobacillus	127	0.731	0.270	0.007	0.017
Paenibacillus	103	0.651	0.316	0.040	0.076
Ruminococcus	38	-1.650	0.329	<0.001	<0.001
Caulobacter	27	2.444	0.438	<0.001	<0.001
Aeromonas	24	-5.661	1.353	<0.001	<0.001
Treponema	22	-1.386	0.492	0.005	0.013

*Normalized reads (hit counts). Only the most abundant genera (>0.01% of all reads) that were altered by the dietary starch source are presented (*n* = 8 per diet and gut site).

[†] Standard error of the $\log_2$fold change.

[‡] False discovery rate (Benjamini-Hochberg) corrected *p* value.

Table S5 Taxonomic identity of *amyA* genes being differently enriched in cecal and colonic samples of CON- and TGS-fed pigs

Taxonomic identity	Mean*	$\log_2$ fold change	SE[†]	*p* value	*q* value[‡]
Cecum					
Bacillus	168	-3.290	0.962	0.001	0.002
Aeromonas	157	-8.451	1.283	<0.001	<0.001
Streptococcus	146	-3.608	0.902	<0.001	<0.001
Butyrivibrio	107	-4.806	1.248	<0.001	0.001
Clostridium	103	-4.964	0.939	<0.001	<0.001
Vibrio	68	-3.292	1.012	0.001	0.004
Enterobacter	22	-5.400	1.142	<0.001	<0.001
Lactococcus	21	-3.512	1.253	0.005	0.014
Pseudomonas	17	-6.164	1.534	<0.001	<0.001
Klebsiella	13	-4.192	1.402	0.003	0.008
Colon					
Bacillus	102	-1.616	0.397	<0.001	<0.001
Streptococcus	101	-1.538	0.456	0.001	0.003
Lactobacillus	68	1.384	0.423	0.001	0.003
Aeromonas	66	-8.154	1.161	<0.001	<0.001
Clostridium	59	-1.443	0.530	0.006	0.014
Butyrivibrio	39	-5.427	0.758	<0.001	<0.001
Vibrio	37	-1.685	0.518	0.001	0.003
Eubacterium	14	2.626	0.501	<0.001	<0.001
Enterobacter	13	-3.918	1.079	<0.001	0.001

*Normalized reads (hit counts). Only the most abundant genera (>0.01% of all reads) that were altered by the dietary starch source are presented ($n = 8$ per diet and gut site).

[†] Standard error of the $\log_2$fold change.

[‡] False discovery rate (Benjamini-Hochberg) corrected *p* value.

Table S6 Taxonomic identity of *araA* genes being differently enriched in cecal and colonic samples of CON- and TGS-fed pigs

Taxonomic identity	Mean*	log_2 fold change	SE[†]	*p* value	*q* value[‡]
Cecum					
Prevotella	289	0.989	0.526	0.060	0.097
Lactobacillus	119	3.178	0.626	0.000	0.000
Clostridium	63	-2.485	0.705	0.000	0.004
Bacillus	29	-2.242	0.651	0.001	0.004
Escherichia	21	-1.715	0.819	0.036	0.071
Leuconostoc	15	1.846	0.562	0.001	0.006
Colon					
Lactobacillus	207	4.166	0.537	0.000	0.000
Prevotella	162	0.619	0.313	0.048	0.067
Clostridium	116	-3.373	0.493	0.000	0.000
Bacillus	53	-2.438	0.486	0.000	0.000
Leuconostoc	24	1.770	0.607	0.004	0.006
Escherichia	21	-3.595	0.660	0.000	0.000
Vibrio	8	-7.342	1.056	0.000	0.000
Paenibacillus	6	-6.910	1.037	0.000	0.000

*Normalized reads (hit counts). Only the most abundant genera (>0.01% of all reads) that were altered by the dietary starch source are presented (n = 8 per diet and gut site).

[†] Standard error of the log_2fold change.

[‡] False discovery rate (Benjamini-Hochberg) corrected *p* value.

Table S7 Taxonomic identity of genes predicted to encode proteins within the KEGG pathway ‘lipopolysaccharide biosynthesis’ differently enriched in cecal and colonic samples of CON- and TGS-fed pigs

Taxonomic identity	Mean*	$\log_2$ fold change	SE[†]	*p* value	*q* value[‡]
Cecum					
Prevotella	4167	2.356	0.459	<0.001	<0.001
Acidaminococcus	519	1.446	0.313	<0.001	<0.001
Veillonella	328	2.491	0.359	<0.001	<0.001
Vibrio	95	-3.297	0.711	<0.001	<0.001
Aeromonas	94	-3.931	0.794	<0.001	<0.001
Porphyromonas	92	-1.640	0.732	0.025	0.075
Tolumonas	85	-5.527	0.876	<0.001	<0.001
Haemophilus	72	-1.674	0.580	0.004	0.020
Akkermansia	70	-4.645	0.905	<0.001	<0.001
Desulfovibrio	50	1.073	0.457	0.019	0.061
Pseudomonas	40	-1.813	0.520	<0.001	0.004
Aliivibrio	40	-2.150	0.800	0.007	0.029
Paludibacter	37	-1.799	0.724	0.013	0.047
Colon					
Prevotella	2848	2.523	0.282	<0.001	<0.001
Bacteroides	1174	0.686	0.260	0.008	0.019
Acidaminococcus	660	1.596	0.329	<0.001	<0.001
Veillonella	450	2.889	0.267	<0.001	<0.001
Parabacteroides	356	-1.771	0.461	<0.001	0.001
Vibrio	125	-3.588	0.768	<0.001	<0.001
Aeromonas	124	-5.885	0.959	<0.001	<0.001
Tolumonas	119	-6.515	0.927	<0.001	<0.001
Shigella	116	-1.904	0.699	0.006	0.015
Helicobacter	109	1.502	0.438	0.001	0.002
Porphyromonas	90	-2.777	0.436	<0.001	<0.001
Haemophilus	72	-2.333	0.664	<0.001	0.002
Wolinella	70	2.263	0.576	<0.001	<0.001
Desulfovibrio	66	1.301	0.371	<0.001	0.002
Flavobacterium	57	-0.961	0.479	0.045	0.081
Pseudomonas	52	-2.414	0.510	<0.001	<0.001
Aliivibrio	42	-2.836	0.899	0.002	0.005
Klebsiella	37	-2.148	0.821	0.009	0.020
Paludibacter	34	-3.288	0.538	<0.001	<0.001
Akkermansia	29	-3.564	0.582	<0.001	<0.001

*Normalized reads (hit counts). Only the most abundant genera (>0.01% of all reads) that were altered by the dietary starch source are presented (*n* = 8 per diet and gut site).

[†] Standard error of the $\log_2$fold change.

[‡] False discovery rate (Benjamini-Hochberg) corrected *p* value.

Table S8 Taxonomic identity of genes predicted to encode proteins within the KEGG pathway 'flagellar assembly' differently enriched in cecal and colonic samples of CON- and TGS-fed pigs

Taxonomic identity	Mean*	$\log_2$ fold change	SE[†]	*p* value	*q* value[‡]
Cecum					
Treponema	2220	-2.200	1.074	0.041	0.091
Aeromonas	1102	-6.425	0.956	<0.001	<0.001
Spirochaeta	480	-2.275	1.120	0.042	0.093
Pseudoalteromonas	160	-4.973	1.020	<0.001	<0.001
Pseudomonas	133	-2.772	0.634	<0.001	<0.001
Eubacterium	115	-2.610	0.574	<0.001	<0.001
Brachyspira	93	-2.686	0.866	0.002	0.007
Vibrio	89	-5.062	0.989	<0.001	<0.001
Alteromonas	83	-6.161	1.088	<0.001	<0.001
Colon					
Aeromonas	1491	-8.233	0.989	<0.001	<0.001
Treponema	713	-5.440	0.642	<0.001	<0.001
Escherichia	568	-3.274	0.909	<0.001	<0.001
Vibrio	313	-7.295	1.110	<0.001	<0.001
Clostridium	300	1.417	0.296	<0.001	<0.001
Campylobacter	176	-2.074	0.405	<0.001	<0.001
Spirochaeta	172	-5.504	0.623	<0.001	<0.001
Pseudoalteromonas	169	-5.518	0.996	<0.001	<0.001
Shigella	92	-3.295	1.003	0.001	0.003
Pseudomonas	91	-2.541	0.685	<0.001	0.001
Heliobacterium	90	2.535	0.552	<0.001	<0.001
Eubacterium	83	-2.377	0.370	<0.001	<0.001
Alteromonas	68	-7.979	1.227	<0.001	<0.001

*Normalized reads (hit counts). Only the most abundant genera (>0.01% of all reads) that were altered by the dietary starch source are presented (n = 8 per diet and gut site).

[†] Standard error of the $\log_2$fold change.

[‡] False discovery rate (Benjamini-Hochberg) corrected *p* value.

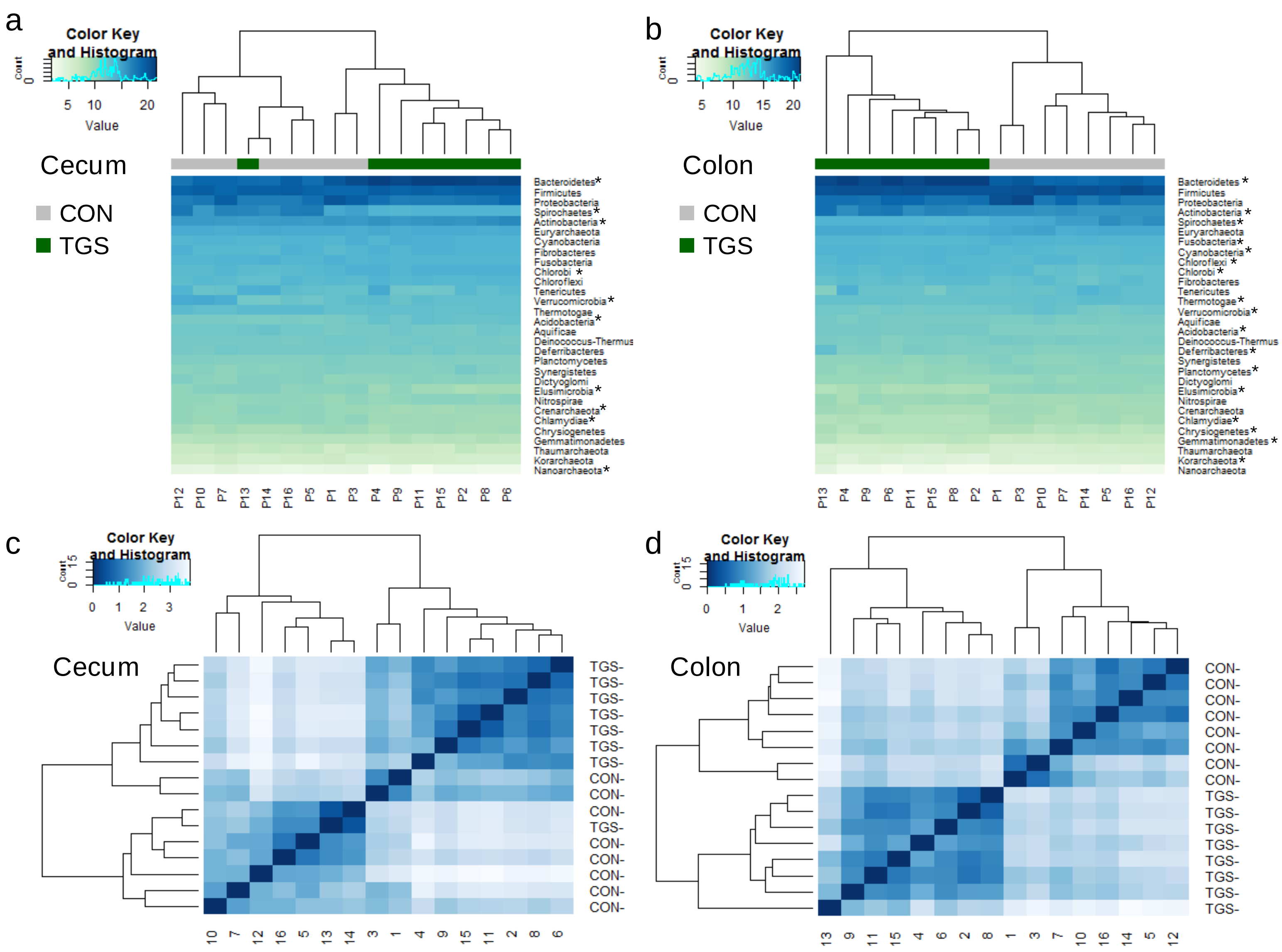
a
b
c
d
Color Key and Histogram
Count
Value
Cecum
Colon
CON
TGS
Bacteroidetes*
Firmicutes
Proteobacteria
Spirochaetes*
Actinobacteria*
Euryarchaeota
Cyanobacteria
Fibrobacteres
Fusobacteria
Chlorobi *
Chloroflexi
Tenericutes
Verrucomicrobia*
Thermotogae
Acidobacteria*
Aquificae
Deinococcus-Thermus
Deferribacteres
Planctomycetes
Synergistetes
Dictyoglomi
Elusimicrobia*
Nitrospirae
Crenarchaeota*
Chlamydiae*
Chrysiogenetes
Gemmatimonadetes
Thaumarchaeota
Korarchaeota
Nanoarchaeota*
TGS-
CON-

Figure S1 Heatmaps of abundances (hit counts) and distance matrices (Euclidian distance) of cecal and colonic samples at phylum level in control starch (CON)- and transglycosylated starch (TGS)-fed pigs (P1-P16). a phyla abundances in cecum; b phyla abundances in colon; c distance matrix of cecal samples; and d distance matrix of colonic samples. Hit counts were normalized with DESeq2-size factors. Abundance change significance between dietary starches is indicated by * (p < 0.05 (false discovery rate, q < 0.10)).

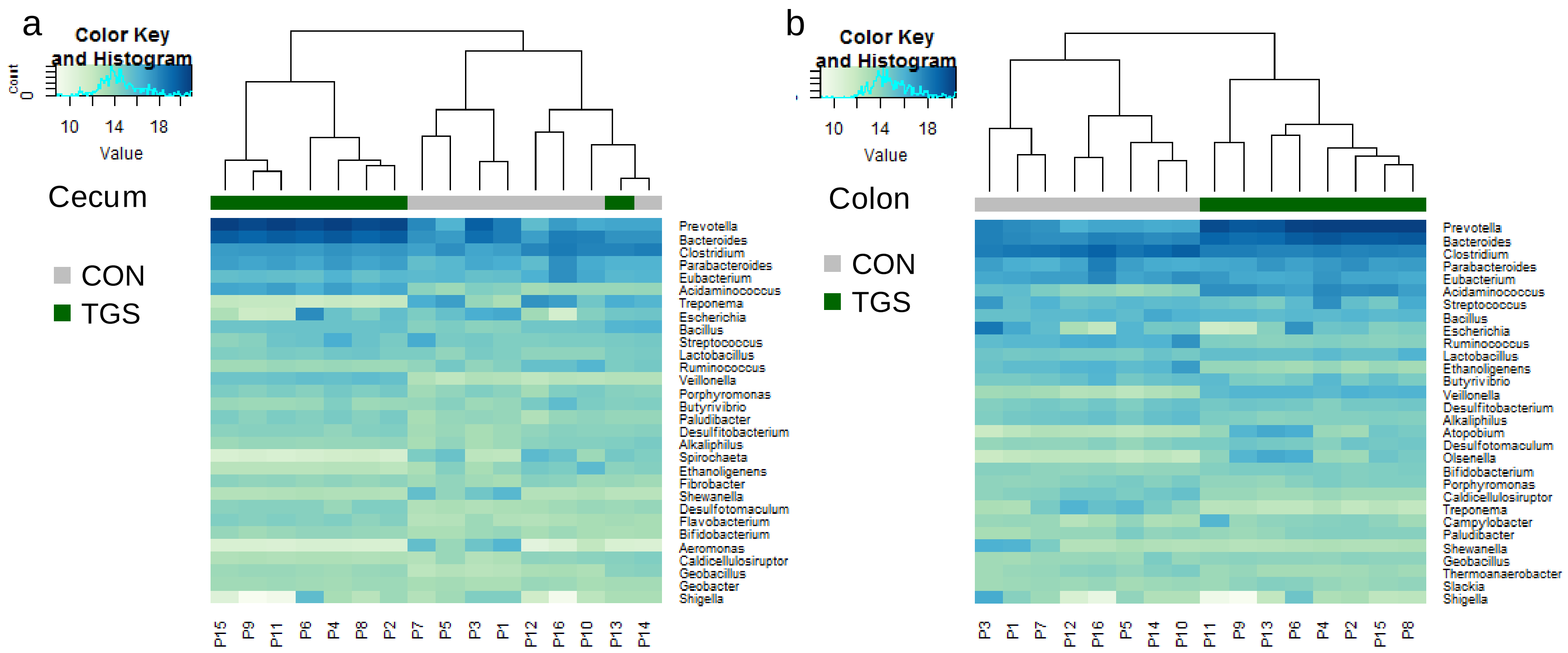

Figure S2 Heatmaps of differently abundant genera (hit counts) in of cecal (a) and colonic samples (b) from control starch (CON)- and transglycosylated starch (TGS)-fed pigs (P1-P16). Only the 30 most abundant genera that were differently affected by the dietary starch source are presented. Hit counts were normalized with DESeq2-size factors. Abundance change significance between dietary starches is $p < 0.05$ (false discovery rate, $q < 0.10$)).
